# Diacetylene‐Functionalized Glycan Mimetics for Receptor‐Mediated Cluster Imprinting in Model Membranes

**DOI:** 10.1002/marc.202500567

**Published:** 2025-10-27

**Authors:** Luca‐Cesare Blawitzki, Lina Charlotte Assenmacher, Nicholas Jäck, Stephan Schmidt, Cornelia Monzel, Laura Hartmann

**Affiliations:** ^1^ Department For Macromolecular Chemistry University of Freiburg Freiburg Im Breisgau Germany; ^2^ Department For Macromolecular Chemistry Heinrich Heine University Düsseldorf Düsseldorf Germany; ^3^ II. Institute of Physics Stuttgart University Stuttgart Germany

**Keywords:** aggregation, clustering, glycocalyx, glycoconjugates, lectin, polydiacetylenes, polymers, vesicles

## Abstract

The glycocalyx, a dense layer of glycoproteins and glycolipids on eukaryotic cells, is essential for cellular functions such as communication, signaling, and pathogen interactions. Certain components spontaneously organize into membrane microdomains, enhancing glycan‐lectin interactions by clustering glycoproteins and glycolipids. However, studying these dynamic systems in native membranes is difficult due to their high heterogeneity. Synthetic glycocalyx mimetics have thus become valuable tools to replicate such complex interactions. In this study, we present diacetylene‐containing multivalent glycomimetic ligands for integration into giant unilamellar vesicles as model membranes. We demonstrate the synthesis and application of a novel SPPoS‐compatible building block that enables site‐selective incorporation of a diacetylene moiety into sequence‐defined, lipidated glycan mimetics. When incorporated into GUVs, the glycomimetic ligands cluster upon lectin binding, bringing diacetylene units into close proximity. UV irradiation then induces polymerization, yielding fluorescent polydiacetylene clusters that mimic receptor‐mediated glycan clustering in cell membranes. This approach allows precise control over glycan cluster formation and provides a versatile platform for studying multivalent glycan‐lectin interactions in clustering and membrane microdomain organization. By stabilizing glycan clusters, this system offers valuable potential for advancing our understanding of membrane‐associated glycan interactions and their role in cellular signaling.

## Graphical abstract

Diacetylene‐containing glycan mimetics cluster upon selective lectin binding in GUVs. Subsequent irradiation leads to the formation of fluorescent polymer clusters, while non‐clustered glycan mimetics remain unaffected in the membrane.

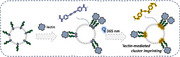

## 1 Introduction

Eukaryotic cells are covered by a dense layer of glycoproteins and glycolipids, which is commonly referred to as the glycocalyx and is involved in a myriad of biological processes such as cellular communication, signaling, and pathogen infection [1, 2]. Membrane‐associated glycans and proteins can be displayed hierarchically organized into local microdomains, termed *lipid rafts*, which cluster membrane proteins and glycoproteins in gel‐state phases of the cell membrane to enhance functional interactions and help cells adapt to changing environments [3, 4]. Similarly, glycans attached to lipids or proteins can be present in the form of dynamically assembled glycan clusters within the fluid plasma membrane, which not necessarily reside within lipid rafts, yielding a high local density of glycan motives, and which are involved in immune recognition, cellular adhesion, and signaling [5, 6]. In fact, this *glycoside cluster effect* can increase the affinity and potentially selectivity of glycan‐lectin interactions and can impact signaling pathways by triggering receptor binding [6, 7, 8]. Furthermore, the spatial arrangement of glycans can create zones that exclude certain molecules, thereby directing cellular behavior and responses [9, 10].

Due to the heterogenous and dynamic nature of native glycocalyces, glycocalyx mimetics have emerged as a valuable tool to recreate simpler cell membrane models [11, 12]. To fully mimic the complexity of biological membranes, it is essential to enhance these synthetic systems by adjusting composition, dynamics, and local carbohydrate heterogeneities. Giant unilamellar vesicles (GUVs) were shown to serve as valuable platforms to mimic various biological processes of the cell membrane [13], such as membrane phase separation [14, 15], cell adhesion [16, 17], or receptor assembly [18]. We recently introduced phase‐separating glycocalyx mimetics that replicate lipid raft formation in native cells [19]. Employing solid phase polymer synthesis (SPPoS), we created lipid‐tethered glycan mimetics, which localize to liquid disordered (Ld) or highly ordered (Lo) membrane domains in GUVs, depending on their respective lipid tether. We observed that lectin binding to ligands in Lo vesicle domains significantly increased compared to Ld domains, emphasizing the role of lipid rafts in enhancing multivalent lectin‐glycan interactions. While this method offers a useful platform to investigate raft‐associated phenomena, the spatial distribution of ligands within these model raft domains is governed predominantly by their intrinsic physicochemical properties. Consequently, dynamic reorganization processes—such as those triggered by receptor engagement—cannot be accurately captured in this system [20, 21]. Thus, introducing dynamically assembled, receptor‐mediated glycan clusters may further refine glycocalyx mimetics to better reflect dynamic responses and local microheterogeneity, while a covalent fixation of such clusters in a controlled manner then facilitates a proper analysis of the effect of cluster formation.

Photocrosslinkers, such as diazirines, aryl azides, and benzophenone‐derivatives, are increasingly being employed in the study of glycan clusters and in identifying binding interactions in native glycocalyces [22, 23, 24]. Yarravarapu et al., for instance, enzymatically functionalized cell‐surface glycans with diazirine‐modified sialic acid to enable the photocrosslinking of glycoproteins [25]. However, photocrosslinkers form covalent bonds with adjacent molecules upon UV irradiation, hence nonspecific crosslinking may obscure the specific binding events of interest [24, 26]. Also, the covalent attachment of a photocrosslinker to the glycan structure and sterically hinder or alter lectin recognition limiting glycan interaction analysis [27, 28].

Previously, Morigaki et al., employed a diacetylene‐containing phospholipid (1,2‐bis(10,12‐tricosadiynoyl)‐sn‐glycero‐3‐phosphocholine, DiynePC) to generate covalently fixed lipid patterns on supported lipid bilayers [29]. The diacetylene moiety is a conjugated 1,3‐diyne that can polymerize upon irradiation with UV light (254 nm) under suitable conditions to give polydiacetylenes exhibiting alternating double and triple bonds, concomitant with an intense coloration due to the extended π‐system [30, 31]. This trigger‐responsive chromism of polydiacetylene makes it ideal as a colorimetric probe, with many studies using head‐group modified diacetylenes as colorimetric sensors for specific metal ions or biomolecules [32, 33, 34]. The polymerization of the diacetylene moieties requires a certain spatial arrangement of the monomers to one another and is therefore termed a topochemical polymerization [35, 36]. Secondary interactions, such as hydrophobic interactions, hydrogen bonding, or π‐stacking can facilitate the appropriate alignment of the diacetylene monomers [37, 38, 39]. Additionally, diacetylenes preferably react with adjacent diacetylenes under optimal packing conditions, hence minimizing unspecific crosslinking with adjacent entities [40].

To generate the patterned lipid membranes, Morigaki et al. prepared supported lipid bilayers of DiynePC via the Langmuir‐Blodgett/Langmuir‐Schäfer technique and subsequently polymerized the aligned diacetylene monomers, employing a photomask and UV‐irradiation. After removal of the non‐polymerized lipids followed by filling the gaps between polymerized regions with native phospholipids, they obtained patterned phospholipid bilayers featuring immobilized polydiacetylene areas interspersed with fluid phospholipid regions [29].

In our study, we extend the methodology of Morigaki et al. by employing diacetylene‐containing glycan mimetics to generate patterned glycocalyx mimetics via lectin‐mediated cluster imprinting, where multivalent lectins act as scaffolds to template and stabilize the spatial organization of glycan microclusters through specific binding interactions. Therefore, first, we have to derive a solid phase synthesis‐compatible diacetylene moiety to be incorporated into lipid‐tethered glycan mimetics as previously introduced. The resulting amphiphilic ligands are characterized regarding their capacity to self‐assemble in aqueous solution and polymerize upon irradiation. Subsequently, we incorporate our ligands into GUVs to create a simplistic glycocalyx model and incubate the vesicles with a suitable lectin to promote ligand‐receptor‐mediated glycan‐clustering. After cluster formation, irradiation induces polymerization of the glycan‐functionalized diacetylene‐macromonomers into an extended π‐conjugated backbone, resulting in fluorescent polydiacetylene‐clusters. Thus, this process mimics the dynamic receptor‐mediated clustering of glycans in native cellular membranes but now allows for fixation, visualization, and further studying of the formed clusters.

## 2 Results and Discussion

### 2.1 Synthesis of Solid Phase Compatible Diacetylene Building Block

One major limitation of commercially available diacetylenes is the requirement for harsh irradiation conditions to initiate polymerization. Specifically, the diacetylene group necessitates exposure to 254 nm UV light, which risks damaging biological molecules [41, 42]. Moreover, the use of readily available diacetylene‐containing fatty acids and lipids restricts the choice of lipid moieties, a factor that significantly influences the physicochemical properties of the ligands in phospholipid membranes [43].

Zhu et al. recently reported on a diacetylene motif, 4,4'‐(buta‐1,3‐diyne‐1,4‐diyl)dianiline, that exhibits a bathochromically shifted absorption profile for polymerization, due to the adjacent aryl moieties, allowing for telechelic functionalization [44]. The diacetylene precursor was synthesized via Eglinton coupling from p‐ethynylaniline and subsequently underwent asymmetric functionalization in solution.

The development of a synthetic strategy for a diacetylene‐containing building block compatible with our previously introduced SPPoS of glycan mimetics was challenged by several limitations. The use of an aromatic diamine precursor poses challenges due to the reduced reactivity of the aromatic amine groups, which could lead to lower yields and decreased coupling efficiency during SPPoS [45, 46]. Additionally, the diacetylene group's susceptibility to polymerization in the solid state further raised concerns about the shelf life of the desired building block.

To address these issues, we decided to divide the target diacetylene containing building block (termed DADS, DiAcetylene Diamine Succinic acid) into two distinct fragments—DADS(I) bearing the carboxylic acid, and DADS(II) bearing an Fmoc‐protected aliphatic amine. The full DADS structure can then be formed on‐resin by first coupling DADS(I) utilizing standard SPPoS coupling protocols, followed by alkyne heterodimerization with DADS(II). This approach preserves the option for further elongation on the solid phase due to the terminal Fmoc‐protected amine group of DADS(II). Conventionally, conjugated diacetylenes are synthesized through Glaser couplings or similar reactions, which work efficiently for symmetric diacetylenes but produce a mixture of coupling products when applied to asymmetric ones [47]. Therefore, we employed the Cadiot‐Chodkiewicz coupling for selective heterocoupling, which uses a terminal haloalkyne and a terminal alkyne, thus minimizing homodimerization as the haloalkyne cannot undergo homocoupling [48]. Accordingly, DADS(I) will be converted into a haloalkyne to enable efficient coupling with DADS(II) via this method on the solid phase.

**FIGURE 1:**
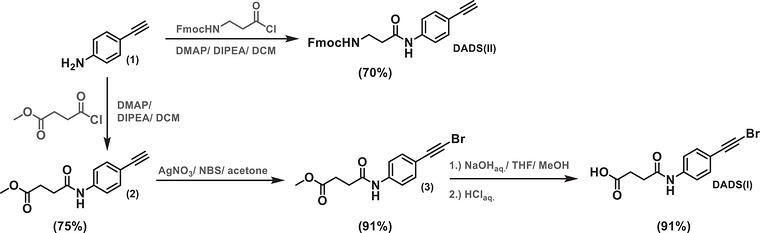
Schematic procedure for the synthesis of DADS building block fragments.

The synthesis of both fragments originated from commercially available p‐ethynylaniline (1). For DADS(I), the aromatic amine was converted into intermediate (2) with methyl succinyl chloride and catalytic amounts of 4‐dimethylaminopyridine (DMAP). Next, (2) was terminally brominated with *N*‐bromosuccinimide (NBS) and AgNO_3_ in acetone to give the respective haloalkyne (3). Finally, (3) was hydrolyzed under alkaline conditions to give the target building block fragment DADS(I) (Figure 1) (see Supporting Information for analytical data of all reaction steps and further details on the synthetic procedure itself).

For the synthesis of DADS(II), (1) was incubated with Fmoc‐β‐Ala‐Cl and catalytic amounts of DMAP to give the target building block fragment in good yield. The introduction of the β‐alanine introduces an aliphatic and, hence, more nucleophilic *N*‐terminus for a more efficient elongation. In this manner, both fragments were synthesized in acceptable yields (DADS(I) with 62% over three synthetic steps, DADS(II) with 70%) and characterized via ^1^H‐NMR, ^13^C‐NMR, and RP‐HPLC‐MS (see Supporting Information).

To demonstrate the applicability of our building block fragment approach, a test sequence was prepared utilizing TentaGel S RAM as the solid phase (see Supporting Information, section *Test sequences for on‐resin DADS synthesis and compatibility with SPPoS*). After sequentially coupling one ethylene glycol diamine succinyl (EDS) as a hydrophilic spacer and DADS(I) to the resin following standard amide coupling protocols, we proceeded with the Cadiot‐Chodkiewicz coupling of the DADS fragments on solid support. As this is, to the best of our knowledge, the first reporting of this reaction on‐resin, the reaction conditions were adopted from established in solution procedures and modified accordingly. After the successful heterodimerization, subsequent Fmoc‐deprotection and further conjugation with another EDS unit proceeded quantitatively, giving the sequence EDS‐DADS‐EDS‐Fmoc in a relative purity >95%, demonstrating the feasibility of our fragment approach.

As our main goal was to synthesize diacetylene‐functionalized, lipidated glycan‐mimetics, we had to test the orthogonality of the DADS moiety toward copper(I)‐catalyzed alkyne‐azide cycloaddition (CuAAC), a method commonly utilized for the coupling of azido‐functionalized sugars to alkynes in SPPoS. Accordingly, the test sequence was subsequently elongated with 4‐pentynoic acid before glycosylation with azido‐functionalized Mannose (Man). RP‐HPLC‐MS analysis indicated the predominant formation of the target product. However, we also observed the formation of a side product resulting from the cleavage of the diacetylene moiety between the conjugated triple bonds. This cleavage regenerates an *N*‐terminal alkyne on the solid support that subsequently undergoes CuAAC in the presence of azido‐functionalized Man, leading to triazole formation. While this demonstrates the general applicability of the fragment approach to incorporate diacetylene moieties in a sequence‐defined manner into a synthetic scaffold via SPPoS, it is noteworthy that the diacetylene moiety is probably not stable under our employed CuAAC conditions and, consequently, for the synthesis of the desired structures has to be installed after glycosylation reactions with CuAAC.

### 2.2 Synthesis of Diacetylene‐Functionalized, Lipidated Glycan‐Mimetics

For the synthesis of the desired lipidated and diacetylene‐bearing glycan mimetics, we employed previously established SPPoS protocols [11, 49]. The use of tailor‐made functional building blocks allows for an iterative assembly of the scaffold, thereby enabling precise control over positioning, density, and valency of the glycan motifs as well as other functional handles, such as the diacetylene moiety and the membrane tether.

**FIGURE 2:**
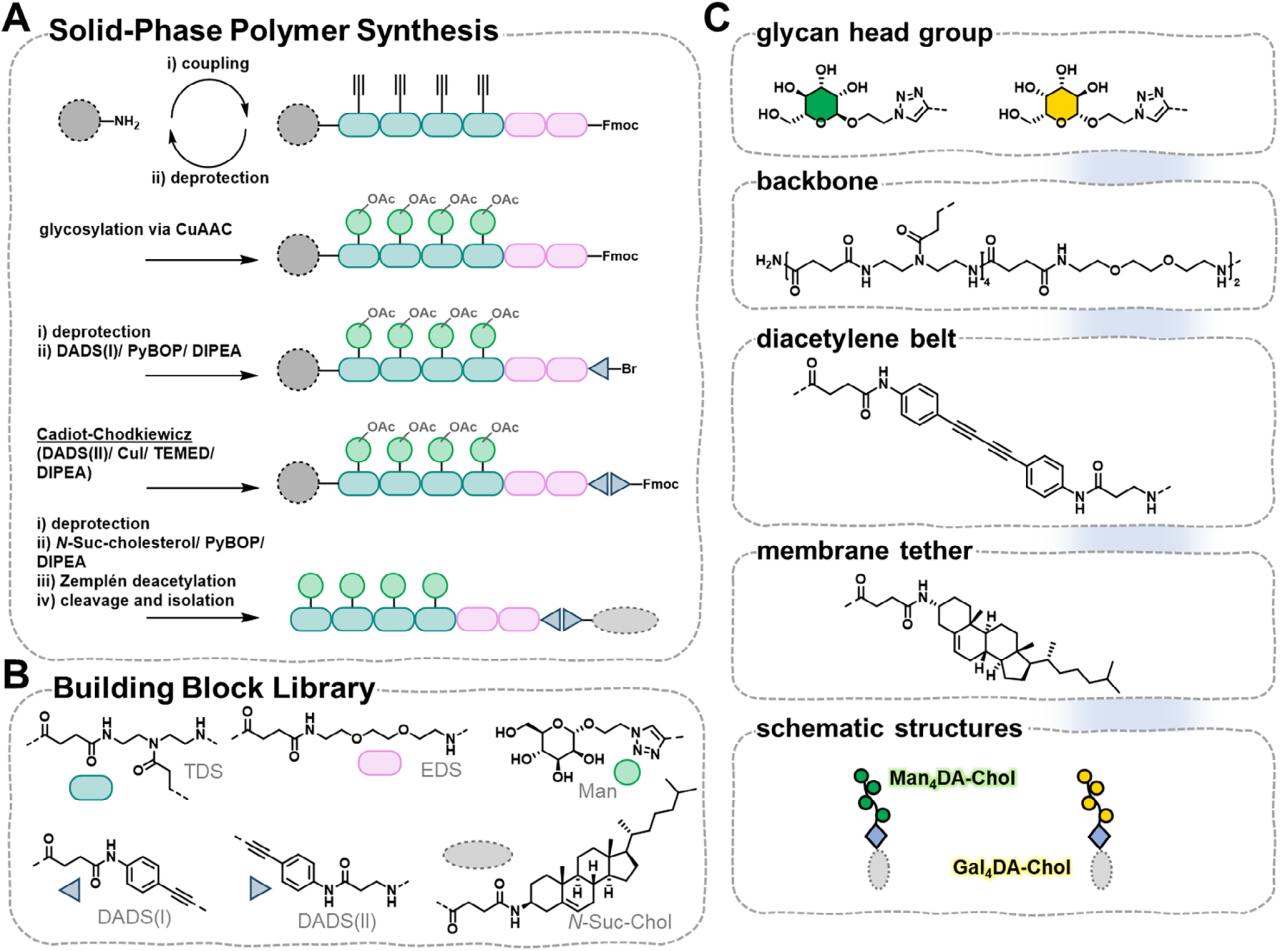
(A) Schematic SPPoS of Man_4_DA‐Chol. (B) Library of employed building blocks. (C) Chemical and schematic structures of the synthesized ligands Man_4_DA‐Chol and Gal_4_DA‐Chol.

The synthesis of the ligands is depicted in Figure 2A. The assembly of the backbone started from commercially available TentaGel S RAM resin, employing iterative assembly of building blocks bearing a free carboxylic acid and an Fmoc‐protected primary amine via stepwise Fmoc‐deprotection and coupling procedures. Four triple bond diethylenetriamine succinyl (TDS) building blocks, carrying an alkyne functionality in their side chain for later glycosylation via CuAAC were installed followed by introducing two EDS units as hydrophilic spacer. After assembly of the scaffold, CuAAC was employed to functionalize the backbone with either Man or galactose (Gal) via their respective acetylated and azido‐functionalized derivatives. Subsequently, the *N*‐terminus was deprotected and functionalized with the DADS(I) fragment, before conjugating the DADS(II) fragment via on‐resin Cadiot‐Chodkiewicz coupling. Finally, the solid‐supported macromolecule was *N*‐terminally functionalized with *N*‐Suc‐3‐β‐aminocholesterol. Unlike in our previous studies, we omitted the use of 3‐β‐azidocholesterol, since the required CuAAC for conjugation leads to partial cleavage of the diacetylene moiety, as described above. After the complete assembly of the polymerizable glycan mimetic, the carbohydrate moieties were deprotected under Zemplén conditions, the structure was cleaved off the resin, collected via precipitation, and further purified by diafiltration.

In this manner, two different glycan mimetics were synthesized, Man_4_DA‐Chol and Gal_4_DA‐Chol, each comprising a tetravalent glyco‐head group (Man or Gal), a polymerizable diacetylene‐moiety, and a cholesteryl‐moiety for subsequent membrane anchoring (Figure 2C). Both structures were confirmed by RP‐HPLC‐MS, ^1^H‐NMR, and HR‐ESI‐MS (see Supporting Information).

### 2.3 Self‐Assembly and Polymerization Behavior of Diacetylene‐Containing Glycan Mimetics

After the successful synthesis of the diacetylene‐functionalized glycan mimetics, we studied the ability of the derived ligands to self‐assemble and to polymerize upon irradiation. We chose Man_4_DA‐Chol as model compound, since both ligands only differ in their respective glycan head group and, hence, the physico‐chemical properties of the two ligands should be very similar.

**FIGURE 3:**
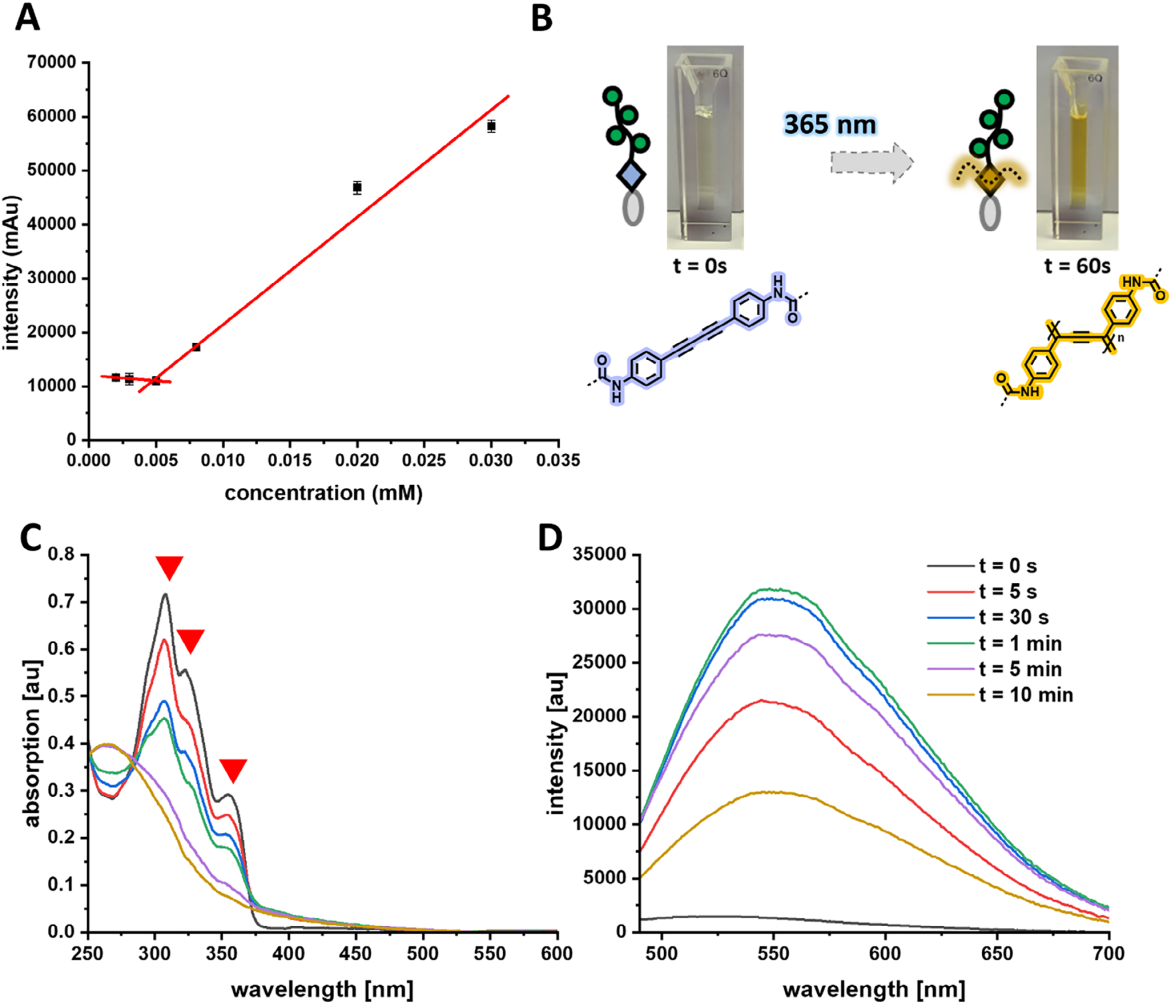
(A) Determination of the CMC for Man_4_DA‐Chol via Nile Red method. Measurements were performed in triplicates, and data points are presented as mean + SEM. (B) Schematic depiction of the crosslinking mechanism upon irradiation, concomitant with an amberish coloration due to an elongated π‐system. (C) The absorption spectrum of the diacetylene moiety shows three absorption maxima at 305, 320, and 355 nm (red arrows). Upon irradiation with an LED spotlight (365 nm, approximately 285 mW/cm^2^) the absorption intensity decreases due to the formation of a polymer network. (D) Upon irradiation, the emission intensity of the diacetylene moiety increases gradually (with a maximum of approximately 550 nm) up to 1 min and decreases afterward, putatively due to photobleaching.

First, we determined the critical micellar concentration (CMC) of Man_4_DA‐Chol employing Nile red as fluorescence read‐out, as was described before [50, 51]. Therefore, a concentration series in deionized water was prepared, and the fluorescence intensity was measured. The gathered fluorescence intensity values were then plotted against the concentration of the ligand. The CMC was determined as the cross‐section of the two linear fits before and after the steep fluorescence increase, yielding a CMC of approximately 5 µm (cp. Figure 3A). In a recent study, we characterized similar amphiphilic structures containing identical sugar head groups and an aromatic moiety in the backbone, though lacking the diacetylene unit and featuring a different apolar tail group. The CMC values for those structures were found to be in a comparable range to the CMC measured in this study [51].

Next, we analyzed the polymerization properties of the ligand upon irradiation. Therefore, we prepared a 0.2 mm solution of the ligand in deionized water (c > cmc) and measured its absorption profile (λ = 250–600 nm), as well as its emission profile (λ_ex_ = 450 nm). The non‐polymerized diacetylene macromolecules exhibit a pronounced absorption profile in the range of 275–375 nm with three absorption maxima at λ = 305 nm, 320, and 355 nm (cp. Figure 3C), which is in accordance with the findings of Zhu et al. [44]. Upon irradiation with 365 nm, the absorption profile decreases over time. Similarly, the emission profile increases gradually with a maximum fluorescence after 1 min of irradiation and a maximum emission wavelength of λ = 550 nm (cp. Figure 3D). Upon prolonged irradiation, the emission profile begins to decrease, putatively due to photochemical damage to the π‐backbone of the polydiacetylene. In addition, the color change of the solution from clear to bright yellow upon UV exposure further indicates successful polymerization and the formation of an extended conjugated structure, allowing for visible light absorption (cp. Figure 3B) [44]. Moreover, size exclusion chromatography indicated successful polymerization of the ligand, as only the irradiated sample showed a distinct peak in the chromatogram, while the non‐irradiated sample showed no detectable signal (see Figure S30).

**FIGURE 4:**
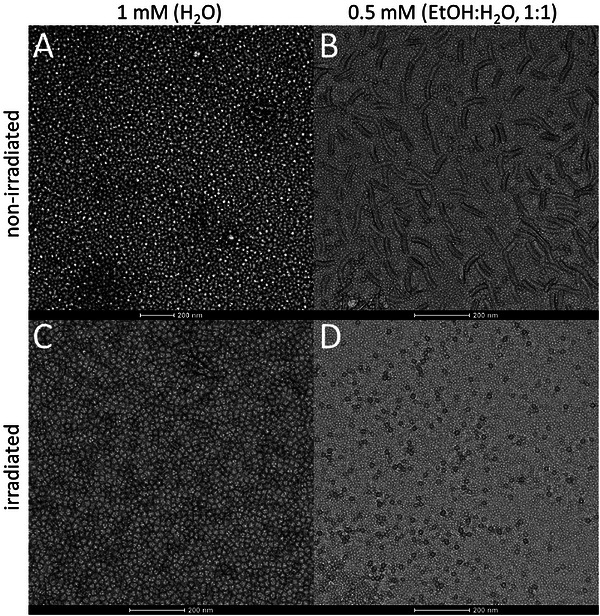
TEM images of the irradiated and non‐irradiated micellar assemblies. At c = 1 mm the ligands form spherical micelles in water (A,C). Upon dilution with EtOH, the crosslinked micelles remain spherical (D), while the non‐crosslinked micelles rearrange into worm‐like micelles (B).

To further investigate the self‐assembling properties of our ligands, we performed TEM imaging, focusing on the morphology of micelles formed in aqueous solutions and their stability in organic solvents, both before and after polymerization. As shown in Figure 4A (before polymerization) and Figure 4C (after polymerization), spherical micelles are formed in ultra‐pure water at a concentration of 1 mm (c > CMC) with a homogenous size distribution. To assess the stability of these micelles, both samples were diluted with ethanol (1:1, v/v), a solvent known to disrupt micelle formation in similar amphiphilic ligands [50]. Notably, the irradiated micelles retained their spherical morphology even after ethanol dilution (Figure 4D), indicating enhanced stability through polymerization.

Interestingly, the non‐irradiated sample also formed micellar aggregates after dilution. However, the morphology shifted markedly from spherical aggregates to larger, worm‐like micelles, suggesting a molecular rearrangement induced by ethanol dilution (Figure 4B). This can be attributed, at least in part, to the TEM preparation and drying process. During sample drying, ethanol tends to evaporate first, which at low ethanol concentrations can promote aggregation. This likely drives the formation of larger, worm‐like structures as a result of molecular rearrangement to limit solvent exposure. Without polymerization, the micelles remain dynamic and prone to reorganization, resulting in the observed change in morphology during the drying phase. In contrast, the irradiated (i.e. polymerized) sample retains the spherical structure, suggesting increased stability and resistance to ethanol‐induced rearrangement.

### 2.4 Lectin‐Mediated Microdomain Formation and Fixation From Diacetylene‐Modified Glycan Mimetics in Model Membranes

Building on the general applicability of our crosslinkable glycan mimetics, we next explored their use in GUVs as model membranes to study glycan clustering through lectin‐mediated ligand recruitment. Therefore, we incorporated Man_4_DA‐Chol into GUVs prepared by electroformation using a matrix consisting of DOPC:Cholesterol (7:3) (2 mg/mL) and 5 mol% of the Man_4_DA‐Chol ligand. We intentionally omitted the addition of a membrane dye to avoid fluorescence crosstalk with the emission signal of the polydiacetylenes.

To first demonstrate the selectivity and specificity of the lectin‐ligand interaction, GUVs were incubated with 200 nM of fluorescently labeled derivatives of Concanavalin A (Con A, Rhodamine B) and Ricinus Communis Agglutinin (RCA, FITC) for 30 min before imaging via fluorescence microscopy. Both, Con A and RCA, are multivalent lectins commonly employed in studying glycan interactions, with Con A having high specificity toward α‐Man residues and RCA having high specificity toward β‐Gal residues.

**FIGURE 5:**
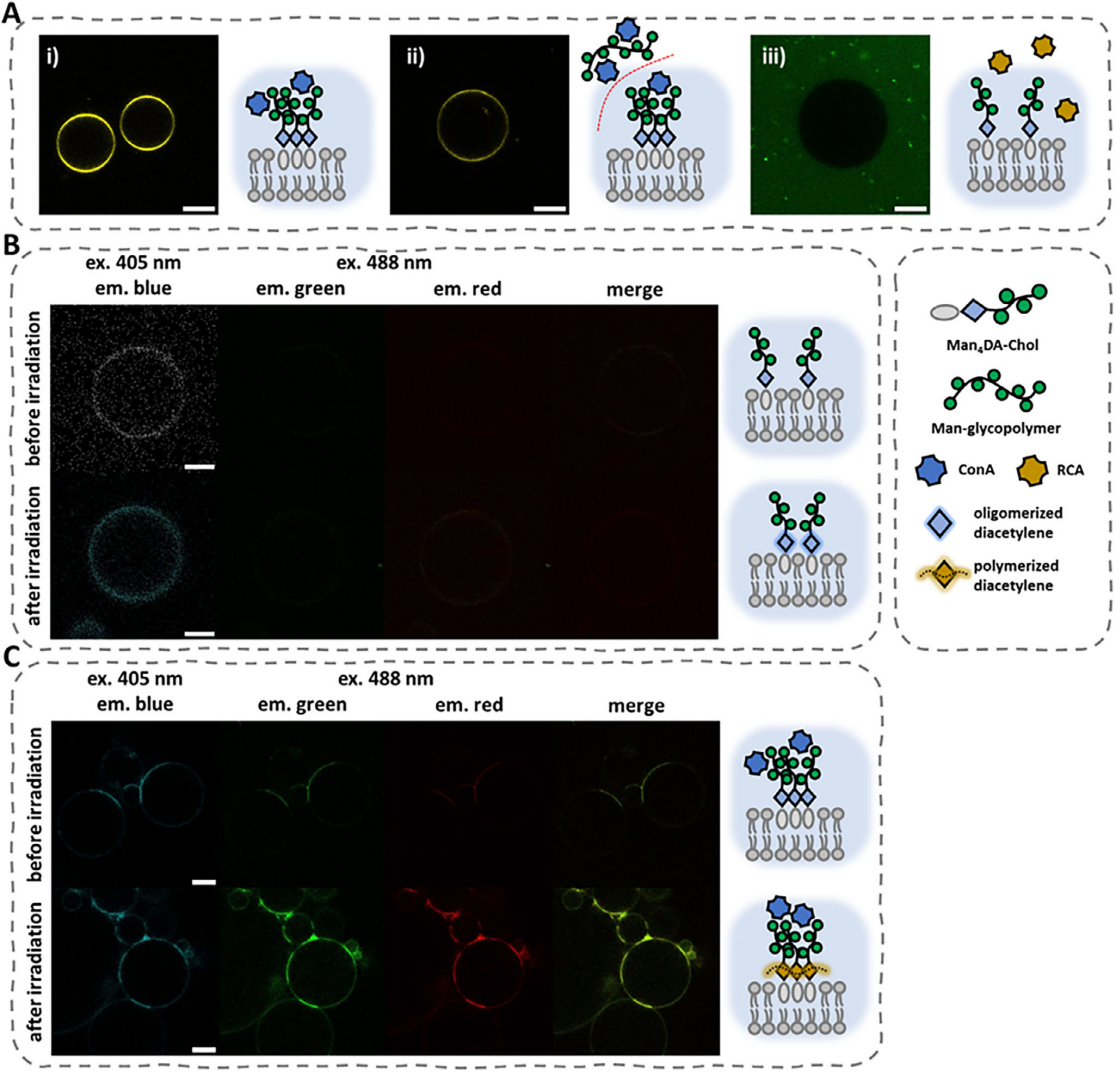
(A) Fluorescently labeled ConA binds to Man_4_DA‐Chol decorated GUVs (i). Due to the dense, multivalent presentation of ligands, inhibition‐competition with a multivalent Man‐polymer cannot completely inhibit ConA binding but drastically reduces the amount of bound lectin (ii). Fluorescently labeled RCA shows no interaction with Man_4_DA‐Chol‐decorated vesicles (iii). (B) Man_4_DA‐Chol‐decorated GUVs show hardly any fluorescence upon excitation, both before irradiation and after irradiation. (C) GUVs incubated for prolonged time with 200 nM ConA form multivalent aggregates, leading to local accumulations of diacetylene‐ligands in the vesicle contact areas. After irradiation of the aggregated vesicle clusters, fluorescence increases drastically, due to the formation of a conjugated polymer‐backbone. Brightness in B and C adjusted for better visibility; high noise level in brightness‐adjusted images indicates low fluorescence/autofluorescence. Scale bars: 10 µm.

As expected, incubation with Con A led to a homogenous lectin fluorescence along the membrane periphery (Figure 5A‐i), while no interaction of RCA with the Man‐decorated vesicles was observable (Figure 5A‐iii). To further validate the specificity, a competitive inhibition assay was performed employing a multivalent Man‐polymer (Pn = 175, c = 0.15 mm) as inhibitor. While pre‐incubation with this multivalent inhibitor did not completely inhibit Con A‐binding, the fluorescence intensity was markedly decreased compared to the non‐inhibited case (Figure 5A‐ii). We suppose that the incomplete inhibition of lectin fluorescence at such high inhibitor concentrations can be attributed to the large excess of multivalent membrane‐bound ligands, which outcompetes the inhibitor and allows residual binding; nonetheless, this still reflects the expected specificity of the lectin–mannose interaction.

Next, we investigated the polymerization behavior of the vesicles in the absence of lectin upon irradiation. Previous research has shown that the polymerization of diacetylene‐functionalized lipids in GUVs is challenging. While the incorporation of diacetylene‐lipids into vesicles composed of saturated lipids, forming a gel phase state, leads to pore formation and rupturing of the vesicles upon irradiation, no reaction can be observed in liquid phase lipid matrices, even at higher diacetylene concentrations [52]. Vesicles were analyzed before and after exposure to UV light (365 nm, 2 min). Prior to irradiation, fluorescence readout was minimal. After irradiation, a slight increase in fluorescence was observed in the UV channel (Figure 5B). This may be attributed to a small fraction of ligands reacting with each other in a diffusion‐limited and statistically random manner, leading to a modest enhancement of UV emission. However, no fluorescence was detected in higher wavelength channels, suggesting that in the absence of ligand clustering, no spatial pre‐organization of the diacetylene ligands and, hence, no significant polymerization occurs, which is in accordance with the literature [52].

Previous work by Römer et al. demonstrated that glycan‐decorated vesicles incubated with multivalent lectins gradually aggregate over time, with lectins locally concentrating glyco‐ligands at the contact sites between vesicles [53]. We aimed to exploit this principle to bring a sufficient number of diacetylene ligands into close proximity, thereby enhancing the efficiency of photoinduced topochemical polymerization. Therefore, Man‐functionalized vesicles were incubated with 200 nm Con A for 2 h to allow the formation of multivalent vesicle aggregates. Of note, fluorescently labeled Con A was deliberately omitted in these experiments to avoid fluorescence crosstalk with the emission signal of the polymerized diacetylenes. The vesicles were imaged before and after irradiation with 365 nm for 2 min.

Prior to UV irradiation, a weak fluorescence signal was detectable in the blue, green, and red channels (Figure 5C). Notably, this initial fluorescence was predominantly localized at sites where vesicles were aggregated by lectins, indicating regions of glycan clustering. We hypothesize that this weak pre‐irradiation signal arises from partial polymerization triggered by ambient light or excitation with the 405 nm laser. The observed localization suggests that the favorable topochemical parameters provided by lectin‐mediated glycan clustering enable spatial alignment of the diacetylene groups, making them susceptible to even low‐intensity irradiation. Nevertheless, in the absence of lectin—and thus in the absence of clustering—no fluorescence was observed prior to UV irradiation, supporting the idea that topochemical preorganization is essential for initiating the polymerization process.

UV irradiation for 2 min led to a pronounced fluorescence increase in all channels, resulting from the polymerization of pre‐clustered diacetylene macromonomers via Con A‐mediated cluster imprinting (Figure 5C).

The enhanced emission signal observed in all three fluorescence channels (blue, green, and red) can likely be attributed to different degrees of polymerization. While shorter polydiacetylene backbones possess smaller conjugated π‐systems and therefore emit at shorter wavelengths, the gradual shift from green to red indicates higher degrees of polymerization and thus longer conjugated π‐systems, which are associated with bathochromically shifted emission [54]. It is also conceivable that rotational twists (i.e., mechanical defects) along the conjugated π‐systems disrupt conjugation along the backbone, resulting in a distribution of π‐systems of different effective lengths, which in turn give rise to emissions at different wavelengths.

As was expected by the precedent inhibition‐competition experiment, inhibition of the Man‐Con A interaction could not be demonstrated under the tested conditions. Neither incubation with monovalent methyl‐α‐D‐mannopyranoside (MeMan) nor with the multivalent Man‐functionalized polymer resulted in the disassembly of the vesicle aggregates. Similarly, treatment with EDTA, a commonly used chelator for sequestering calcium ions essential for the activity of C‐type lectins such as Con A, or trypsinization at 37°C did not lead to cluster dissociation (data not shown).

Photoinduced polymerization of diacetylenes is supposed to generate reactive radical intermediates. In addition, Con A contains aromatic residues that can undergo photo‐induced radical generation. Based on our observation that Con A binding to the polymeric assemblies could not be reversed, we hypothesized that Con A might have been covalently incorporated into the polymer network via radical‐mediated crosslinking. To investigate this possibility, micellar assemblies of Man^4^DA‐Chol were incubated with Con A for 60 min. Subsequently, one portion of the sample was irradiated, while another was kept non‐irradiated as a control, and both were analyzed by MALDI‐TOF MS to probe potential protein–polymer linkage, rather than the formation of polydiacetylene itself. In all cases, the main mass signal at 25.6 kDa corresponded to monomeric Con A, consistent with tetramer dissociation under MALDI conditions. Importantly, no additional mass signals were detected after irradiation, indicating that covalent crosslinking of Con A under the applied polymerization conditions did not occur (see Figure S34).

We thus hypothesize that the high avidity of the multivalent interaction between the surface‐displayed ligands and Con A, combined with the resulting steric shielding of the binding sites, prevents effective inhibition or degradation. As a consequence, the vesicle clusters remain stable despite the presence of competitive binders, calcium chelation, or enzymatic digestion.

## 3 Conclusion

We have developed a novel building block compatible with SPPoS that enables the site‐selective incorporation of a polymerizable diacetylene moiety into glycan mimetics. This was achieved through an efficient on‐resin Cadiot–Chodkiewicz coupling between two building block fragments, resulting in high conversion rates without side‐product formation. Using this strategy, we synthesized diacetylene‐functionalized glycan mimetics bearing tetravalent Man or Gal head groups and a cholesteryl anchor for membrane tethering. The behavior of the diacetylene‐functionalized glycan mimetics in solution was investigated by monitoring changes in their absorption and emission spectra upon irradiation, which are indicative of photo‐induced polymerization. Additionally, transmission electron microscopy (TEM) was used to assess their morphological stability: after irradiation, the micelles retained their spherical shape, consistent with successful polymerization, whereas non‐irradiated micelles exhibited morphological changes upon ethanol dilution, suggesting that polymerization stabilizes their supramolecular structure.

When incorporated into giant unilamellar vesicles (GUVs), the Man‐functionalized ligand enabled the formation of simplified glycocalyx mimetics. Selectivity and specificity of the system were confirmed by control experiments: While incubation with fluorescently labeled Con A led to a homogenous fluorescence along the vesicle membrane, incubation with fluorescent Ricinus communis agglutinin (RCA), a galactose‐binding lectin, did not lead to any interactions of the lectin with the Man‐decorated vesicles. Pre‐incubation of Con A with a multivalent mannose‐based inhibitor was shown to minimize Con A binding to the Man‐decorated vesicles, further demonstrating the selectivity of the system.

Polymerization of the diacetylene‐ligands did not occur in the absence of a suitable receptor, indicating that the diacetylene moieties were not in sufficient proximity to undergo topochemical polymerization under these conditions. However, upon incubation with the Man‐specific lectin Con A, multivalent clustering of the ligands within the membrane was induced, which enabled spatial pre‐organization, and subsequent UV‐induced polymerization yielded the formation of fluorescent glycan mimetic microdomains, a mechanism that we termed lectin‐mediated cluster imprinting. Attempts to disassemble the Con A‐induced clusters using monovalent ligands, trypsinization or calcium chelation with EDTA were yet unsuccessful, likely due to the high avidity of the multivalent interactions and steric shielding of the binding sites.

Altogether, this work introduces a versatile strategy for the spatial and temporal control of glycan clustering in glycocalyx mimetics. We envision that these novel glycan mimetics will serve as valuable tools to investigate dynamic lectin–glycan interactions, including those involved in host–pathogen recognition, and to study how pre‐clustered architectures modulate biological responses in the glycocalyx.

## Funding

Deutsche Forschungsgemeinschaft. Grant Numbers: CRC 1208 A11, CRC 1208 A12, CRC 1535 A09, Volkswagen Foundation. Grant Number: Freigeist‐Fellowship and Momentum (project number 0072536‐00), European Research Council. Grant Number: 101088228.

## Conflicts of Interest

The authors declare no conflicts of interest.

## Supporting information




**Supporting File**: marc70108‐sup‐0001‐SuppMat.pdf.

## Data Availability

The data that support the findings of this study are available in the supplementary material of this article.
