# Supplementary material for: Diacetylene‐Functionalized Glycan Mimetics for Receptor‐Mediated Cluster Imprinting in Model Membranes
Source: Macromol Rapid Commun. 2025 Oct 27;47(14):e00567. doi: 10.1002/marc.202500567 (PMC13384804; doi:10.1002/marc.202500567)
Supplement: Supplementary file 1 — Supporting File: marc70108‐sup‐0001‐SuppMat.pdf. [file MARC-47-e00567-s001.pdf]

*Supplementary Information*

# Diacetylene-Functionalized Glycan Mimetics for Receptor-Mediated Cluster Imprinting in Model Membranes

*Luca-Cesare Blawitzki[a,b], Lina Charlotte Assenmacher[b], Nicholas Jäck[a], Stephan Schmidt[a], Cornelia Monzel[c,d], Laura Hartmann[a]*

[a] Department for Macromolecular Chemistry, University of Freiburg, Freiburg im Breisgau, Germany

[b] Department for Macromolecular Chemistry, Heinrich Heine University Düsseldorf, Düsseldorf, Germany

[c] Department for Experimental Medical Physics, Heinrich Heine University Düsseldorf, Düsseldorf, Germany

[d] present address: 2nd Institute of Physics, University of Stuttgart, Stuttgart, Germany

*Email: laura.hartmann@makro.uni-freiburg.de

# Table of Contents

Materials ........ 3
Instrumentation ........ 4
General Methods ........ 6
Experimental Section ........ 8
Analytical Data ........ 11
Test sequences for on-resin DADS synthesis and compatibility with SPPoS ........ 17
Experimental ........ 31
References ........ 36

# Materials

D-(+)-galactose, D-(+)-mannose, N,N-dimethylformamide, oxalyl chloride, piperidine and tosyl chloride were purchased from Acros Organics. Ethyl trifluoroacetate and PyBOP were purchased from Apollo Scientific. Acetonitrile and cholesterol were purchased from AppliChem. Fmoc-Cl and p-ethynylaniline were purchased from bld pharm. Succinic anhydride was purchased from Carbolution Chemicals. DIPEA and sodium chloride were purchased from Carl Roth. 1.4-dioxane, acetic anhydride, calcium chloride, ethyl acetate, HEPES, n-hexane, hydrochloric acid (37%), manganese dichloride, potassium carbonate, sodium azide, sodium bicarbonate, sodium hydroxide, sodium methoxide and trimethylamine were purchased from Fisher Scientific. Triethylsilane was purchased from fluorochem. Fmoc-β-Ala-OH was purchased from Iris Biotech. Concanavalin A was purchased from MP Biomedicals. 2,2'-(Ethylenedioxy)bis(ethylamine), 2-bromoethanol, 4-pentynoic acid, boron trifluoride diethyl etherate, Bovine Serum Albumin, dichloromethane, diethyl ether, diethylenetriamine, formic acid, methanol, tetrahydrofuran, trifluoroacetic acid, NBS, silver nitrate and triisopropylsilane were purchased from Sigma-Aldrich. Trimethylsilyl azide and trityl chloride were purchased from TCI chemicals. Methyl succinyl chloride and sodium diethyldithiocarbamate were purchased from Thermo Scientific.

All solvents and reagents used were purchased in the highest purity available and used without further purification.

TentaGel® S RAM resin (loading: 0.26 mmol/g) was purchased from RAPP Polymere GmbH.

DOPC was purchased from Avanti Polar Lipids.

Dialysis was performed via diafiltration in VIVASPIN 20 centrifugal concentrators (MWCO: 10 kDa; PES) from sartorius.

Experiments were conducted in 18-well glass bottom µ-slides from ibidi GmbH.

# Instrumentation

**Reversed Phase- High Pressure Liquid Chromatography- Mass Spectrometry (RP- HPLC-MS)/Electron Spray Ionization- Mass Spectrometry (ESI-MS)**

RP-HPLC-MS was carried out on an Agilent 1260 Infinity instrument coupled to a variable wavelength detector (VWD) (set to 214 nm) and a 6120 Quadrupole LC/MS containing an Electrospray Ionization (ESI) source (operation mode positive, m/z range from 200 to 2000). An MZ-AquaPerfect C18 (3.0 × 50 mm, 3 µm) RP column from MZ-Analysentechnik was used. As eluent system water/acetonitrile containing 0.1 vol% formic acid was applied. The mobile phases A and B were: System A) water/acetonitrile (95/5, v/v); System B) water/acetonitrile (5/95, v/v). The samples were analyzed at a flow rate of 0.4 ml/min using a linear gradient, starting with 100% of system A) and reaching 100% system B) within 17 min. The temperature of the column room was set to 25 °C. All purities were determined using the OpenLab ChemStation software for LC/MS from Agilent Technologies.

### Ultra High Resolution - Mass Spectrometry (UHR-MS)

UHR-MS measurements were performed with a Bruker UHR-QTOF maXis 4G instrument with a direct inlet via syringe pump, an ESI source and a quadrupole followed by a Time of Flight (QTOF) mass analyzer.

### Nuclear Magnetic Resonance Spectroscopy (NMR)

The $^{1}$H-NMR spectra were recorded on a Bruker Avanace III 600 (300 or 600 MHz). These spectra were evaluated according to the following scheme: (frequency in MHz, deuterated solvent), chemical shift in ppm (multiplicity, coupling constant, integral, signal assignment). The chemical shift is given in relation to the $^{1}$H signals of the deuterated solvents used.

### Lyophilization

The final glycomacromolecules were lyophilized with an Alpha 1-4 LD plus instrument from Martin Christ Freeze Dryers GmbH (-40 °C, 0.1 mbar).

### Fluorescence Microscopy

Giant Unilamellar Vesicles were imaged confocally on a Leica DMi8 inverted microscope (Leica Microsystems CMS GmbH, Wetzlar, Germany), equipped with a 63x oil-objective and a Leica DFC9000 GT camera, operating the LAS X software.

Excitation was performed using either a 405 nm laser (100% power) or a 488 nm laser (100% power). Emission was recorded in three channels: blue (430–470 nm, 500% gain), green (530–570 nm, 500% gain), and red (630–700 nm, 1000 V gain). Pinhole 1 Airy.

Images were evaluated via ImageJ 1.54g.

### UV-Vis Spectroscopy

UV-Vis measurements were performed at 25 °C on a dual-trace spectrometer Specord® 210 Plus from Analytik Jena AG (Jena, Germany), using Win ASPECT PLUS software to operate the instrument.

## UV-Irradiation

UV-irradiation of samples was conducted with a UV-LED spot with a wavelength of 365 nm and a max irradiation intensity of 950 mW/cm$^2$. LED-spot and LED control were both purchased from Opsytec Dr. Gröbel.

## Size Exclusion Chromatography-Multi-Angle Light Scattering ($H_2O$-SEC-MALS)

SEC analysis was conducted with an Agilent 1200 series HPLC system and three aqueous SEC columns provided by Polymer Standards Service (PSS). The columns were two Suprema Lux analytical columns (8 mm diameter and 5µm particle size) and one precolumn (50 mm, 2×160 Å of 300 mm and 1000 Å of 300mm). The eluent was a buffer system consisting of MilliQ water and 30% acetonitrile with 50 mM NaH2PO4, 150 mM NaCl and 250 ppm NaN3 with a pH = 7.0 (via addition of 50 mL of 3 molar aqueous sodium hydroxide solution) filtered with an inline 0.1 µm membrane filter and running at 0.8 mL per min. Multi-angle light scattering is S3 recorded via mini DAWNTREOS and differential refractive index spectra with Optilab rEX both supplied by Wyatt Technologies EU. Data analysis was committed with Astra 5 software and a dn/dc value of 0.156 for each polymer.

## MALDI-TOF MS

MALDI-TOF analysis was conducted on a Ultraflex I from Bruker Daltonics. The samples were measured in linear mode, employing 2.5-dihydroxybenzoic acid (DHB) as matrix.

# General Methods

### Solid-Phase Polymer Synthesis

All structures were prepared on Tentagel® S RAM resin (batch size: 0.1 mmol). All washing steps were conducted with 4 mL solvent. Polypropylene reactors equipped with polyethylene frits and closed with Luer-stoppers were used. For the glycomacromolecules, the head-group sequence was first assembled, followed by glycoconjugation via CuAAC (copper(I)-catalyzed alkyne-azide cycloaddition), DADS(I) conjugation, DADS(II) conjugation via Cadiot-Chodkiewicz coupling, terminal Fmoc-deprotection and capping with *N*-Suc-3-β-aminocholesterol.

### General coupling protocol

For all compounds, the resin was first swollen in 4 mL DCM for 30 min, subsequently washed ten times with DMF, Fmoc-deprotected by treating with 25% piperidine in DMF (three times for ten minutes) and again washed fifteen times with DMF. Employed building blocks were coupled to the N-terminus by adding the respective building block (5eq.), PyBOP (5 eq.) and DIPEA (20 eq.) in 4 mL DMF to the resin and shaking for 1h. Then, the resin was washed fifteen times with DMF and the N-terminus was again deprotected followed by the next coupling step.

### Fmoc cleavage

The Fmoc-protecting group of the resin as well as from the coupled building blocks or amino acids was cleaved by means of 4 mL of a 25% solution of piperidine in DMF to release the primary amine. The deprotection was carried out twice for 10 min. Afterwards the resin was washed 10 times with DMF before coupling.

### CuAAC protocol for glycosylation

To the oligomeric structure loaded on the resin 2.5 eq of acetyl protected 2-azidoethyl pyranoside (α-Mannose/ β-Galactose) per alkyne group dissolved in 4 mL DMF were added. Secondly 50 mol% sodium ascorbate per alkyne group and 50 mol% CuSO4 per alkyne group were dissolved each in a small amount of water and also added to the resin. The syringe reactor was rocked for 18 h before the solution was discarded. The resin was washed three times with DMF and subsequently treated with a 23 mM solution of sodium diethyldithiocarbamate in DMF and water (50/50, v/v) and alternating with DMF and DCM until no further color change occured.

### On-resin Cadiot-Chodkiewicz Coupling

3 eq of DADS(II) and 20 eq of DIPEA were dissolved in a mixture of MeOH and DMF (1:1, v:v). 0.1 eq CuI was suspended in little DMF and complexated with 0.1 eq TEMED. The copper and the DADS(II) solution were drawn into the syringe reactor and rocked for 16h. The solution was discarded and remaining copper was removed via treatment with a 23 mM solution of sodium diethyldithiocarbamate in DMF and water (50/50, v/v) and alternating washing steps with DMF and DCM until no further color change occurred.

### Microcleavages

To monitor the iterative assembly of the diacetylene-containing macromolecular backbone on the solid phase, microcleavages were performed. For this purpose, the solid-phase resin was washed five times with DCM, and a small amount of resin was transferred into an Eppendorf tube. The resin was treated with 100 µL of TFA and shaken for 15 minutes. The sample was then precipitated by addition of 1 mL of cold diethyl ether and centrifuged. The supernatant was decanted, and the residue was

dried under a gentle stream of nitrogen. The residue was subsequently dissolved in a 1:1 (v/v) mixture of water and acetonitrile and subjected to RP-HPLC-MS analysis.

### On-resin deacetylation

Glycooligomers were deacetylated under Zemplén conditions. The resin was washed five times with methanol, before 5 mL of a 0.2 M solution of NaOMe in MeOH was drawn into the reactor. The resin was rocked for 30 min, before the solution was discarded. The resin was then washed twice with MeOH before another 5 mL of the methoxide solution were drawn into the reactor. After 30 min, the solution was again discarded and the resin was washed five times with MeOH.

### Cleavage from the solid phase

The resin was washed five times with DCM before acidic cleavage from the resin with a cocktail consisting of TFA, TIPS and DCM (95/2.5/2.5, v/v/v) for 30 min.

The cleavage solution was precipitated in ether. The precipitate was collected via centrifugation, dried under a gentle stream of nitrogen and dialyzed via diafiltration against ultrapure water in five cycles (20 mL each). The dialyzed compounds were dissolved in fresh ultrapure water and lyophilized.

### Preparation of Giant Unilamellar Vesicles (GUVs)

25 µL of a solution of DOPC:Cholesterol (7:3) in $CHCl_3$ (2 mg/mL) were mixed with 5 mol% of $Man_4$DA-Chol (0.4 mg/mL, 20% MeOH in $CHCl_3$) and deposited on cleaned ITO glasses (pgo GmbH, Iserlohn, Germany) and dried under reduced pressure for 1 h in a vacuum oven at 40°C. The swelling chamber was assembled, filled with 1000 µL of a 115 mM sorbitol solution (to match the osmolarity of the measuring buffer (lectin binding buffer (LBB) (10 mM HEPES, 50 mM NaCl, 1 mM MnCl2, 1 mM CaCl2, pH 7.4))), and an AC field was applied to the ITO slides (divided by a Teflon spacer) using a function generator with an amplitude of 2.4 V and a frequency of 89 Hz. Electroformation was carried out for 75 min at ambient temperature before collecting the vesicles.

### Preparation of Measuring Chambers

18-well ibidi glass slides were passivated with BSA (5 mg/ml in ultrapure water) for 20 min. The chambers were rinsed thrice with lectin binding buffer and filled with fresh lectin binding buffer.

### Preparation of MALDI-TOF MS samples

200 µL Man4DA-Chol (200 µM, c > cmc) and 50 µL Con A (100 µM)(both dissolved in LBB) were incubated at ambient temperature for 60 minutes. Afterwards, one sample was irradiated for 2 minutes with 365 nm, while the other one was kept non-irradiated. Both samples were diluted with 250 µL LBB containing 0.2 % TFA. Similarly, 50 µL Con A (100 µM in LBB) was diluted with 200 µL LBB + 250 µL LBB containing 0.2 % TFA.

Samples were analyzed via MALDI-TOF-MS, employing a 2.5-dihydroxybenzoic acid (DHB) matrix.

# Experimental Section

## Synthesis of Building Blocks and Monomers

The building blocks ethylene glycol diamine succinic acid (EDS) and triple bond diethylenetriamine succinic acid (TDS) were prepared as reported earlier.[1] Tetra-O-acetyl-azidoethyl-α-d-mannopyranoside and tetra-O-acetyl-azidoethyl-β-d-galactopyranoside were synthesized according to the literature protocols.[2] N-Suc-3-β-aminocholesterol was synthesized according to a protocol by *Kim et al*.[3] Fmoc-β-Ala-Cl was synthesized according to a protocol by *Song et al*.[4]

## Synthesis of methyl 4-((4-ethynylphenyl)amino)-4-oxobutanoate (2)

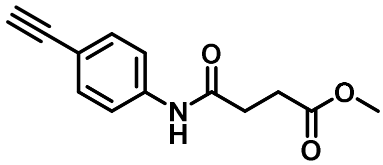


0.5 g 4-Ethinylaniline (4.2 mmol), 5 eq DIPEA and 0.1 eq DMAP were dissolved in 15 mL of DCM and cooled in an ice bath. 1.3 eq monomethylsuccinyl chloride were dissolved in 10 mL of DCM and added dropwise to the solution. The mixture was left to stir for 16 h, thereby allowed to reach ambient temperature. The solution was then washed thrice with 20% citric acid in water, the organic phase was dried over sodium sulfate, filtered and the solvent was removed under reduced pressure.

The crude product was purified via column chromatography (hexanes: ethyl acetate, 1:1, v:v).

$r_f$ = 0.61

yield: 0.73 g of a crystalline, brownish solid (75%)

**$^1$H-NMR (400 MHz, $CDCl_3$) δ [ppm]** = 7.87 (s, 1H, Ar-N*H*), 7.47 (d, *J* = 8.3 Hz, 2H, Ar-*H*), 7.43 – 7.39 (m, 2H, Ar-*H*), 3.70 (s, 1H, -C*H*$_3$), 3.03 (s, 1H, alkyne-*H*), 2.74 (dd, *J* = 7.2, 5.7 Hz, 2H, Ar-NHC(O)C*H*$_2$CH$_2$C(O)), 2.66 (dd, *J* = 7.5, 5.9 Hz, 2H, Ar-NHC(O)CH$_2$C*H*$_2$C(O))

**Synthesis of methyl 4-((4-(bromoethynyl)phenyl)amino)-4-oxobutanoate (3)**

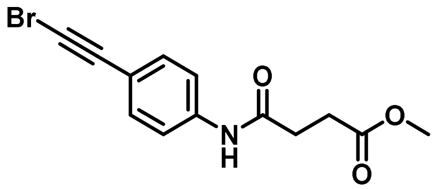

0.5 g **(2)** (2.2 mmol) and 1.1 eq freshly recrystallized NBS were dissolved in 20 mL acetone. The flask was covered in aluminum foil to minimize exposure to light and 0.1 eq AgNO3 was added. The mixture was stirred for 16 h before quenching with 50 mL of a saturated solution of sodium thiosulfate. The aqueous solution was extracted thrice with ethyl acetate, the pooled organic fractions were washed with brine, dried over magnesium sulfate and the solvent was removed under reduced pressure.

The crude product was directly used for the subsequent reaction.

Yield: 0.61 g of a crystalline, amberish solid (91%)

**Synthesis of DADS (I)**

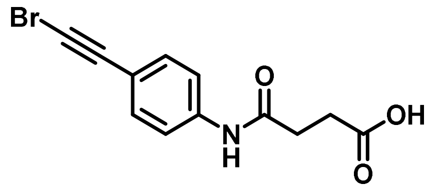

The crude **(3)** (0.61 g, 2.0 mmol) was dissolved in 15 mL of a mixture of methanol, THF and 2 M $NaOH_{aq.}$ (4:4:2, v:v) and stirred for 16 h. The organic solvents were evaporated under reduced pressure, the remaining solution was diluted with 10 mL of water and acidified with cold 0.5 M hydrochloric acid (pH = 3). The aqueous solution was extracted thrice with ethyl acetate, the pooled organic fractions were dried over magnesium sulfate, filtered and the solvent was removed under reduced pressure.

Yield: 0.54 g of a crystalline, sand-colored solid (91%)

**$^{1}$H-NMR (400 MHz, DMSO-d6) δ [ppm]** = 12.13 (s, 1H, -COO*H*), 10.15 (s, 1H, Ar-N*H*-), 7.62 – 7.54 (m, 2H, Ar-*H*), 7.43 – 7.35 (m, 2H, Ar-*H*), 2.61 – 2.45 (m, 4H, C(O)$CH_2CH_2$C(O))

**$^{13}$C-NMR (400 MHz, DMSO-d6) δ [ppm]** = 173.79 (C*4*), 170.43 (C*1*), 139.96 (C*5*), 132.49 (C*7*), 118.74 (C*6*), 115.89 (C*8*), 79.86 (C*9*), 51.29 (C*10*), 31.15 (C*2*), 28.71 (C*3*)

**ESI-MS** calc. for C12H10BrNO3: $[M+1H]^{1+}$ 296.0; found 296.0 $[M+1H]^{1+}$

**RP-HPLC**: $t_R$ = 9.84 min, >98% relative purity (UV), from 95/5 to 5/95 Vol. % Water/acetonitrile with 0,1% formic acid in 20 min at 25 °C.

**Synthesis of DADS(II)**

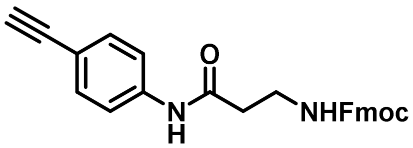

0.5 g p-Ethynylaniline (4.2 mmol), 5 eq DIPEA and 0.1 eq DMAP were dissolved in 20 mL DCM and cooled in an ice bath. 1.2 eq Fmoc-β-Ala-Cl was dissolved in 10 mL DCM and added dropwise to the solution. The mixture was left to stir for 16 h. The solution was then washed with a 20% citric acid solution, twice with brine, dried over magnesium sulfate and evaporated to dryness. The crude product was recrystallized from a solution of 2% MeOH in chloroform.

Yield: 1.2 g of an amorphous, sand-colored solid (70%)

**$^1$H-NMR (400 MHz, DMSO-d6) δ [ppm]** = 10.10 (s, 1H, Ar-N*H*-), 7.86 (d, *J* = 1.0 Hz, 2H, Ar-*H*), 7.69 – 7.56 (m, 4H, Ar-*H*), 7.48 – 7.22 (m, 7H, N*H*C(O)O, Ar-*H*), 4.33 – 4.13 (m, 3H, N*H*C(O)OC*H*$_2$C*H*-), 4.05 (s, 1H, alkyne-*H*), 3.27 (t, *J* = 6.4 Hz, 2H, Ar-NHC(O)CH$_2$C*H*$_2$NH), 2.54 – 2.48 (m, 2H, Ar-NHC(O)C*H*$_2$CH$_2$NH)

**$^{13}$C-NMR (400 MHz, DMSO-d6) δ [ppm]** = 169.52 (C*7*), 156.05 (C*10*), 143.89 (C*13*), 140.72 (C*18*), 139.63 (C*6*), 132.33 (C*4*), 127.59 (C*16*), 127.04 (C*17*), 125.16 (C*15*), 120.09 (C*5*), 118.83 (C*14*), 115.96 (C*3*), 83.61 (C*2*), 79.73 (C*1*), 65.37 (C*11*), 46.69 (C*12*), 36.75 (C*8*), 36.63 (C*9*)

**ESI-MS** calc. for C12H10BrNO3: $[M+1H]^{1+}$ 411.2; found 411.2 $[M+1H]^{1+}$

**RP-HPLC**: $t_R$ = 13.23 min, >98% relative purity (UV), from 95/5 to 5/95 Vol. % Water/acetonitrile with 0,1% formic acid in 20 min at 25 °C.

# Analytical Data

N-Suc-4-ethynylaniline methyl ester **(2)**

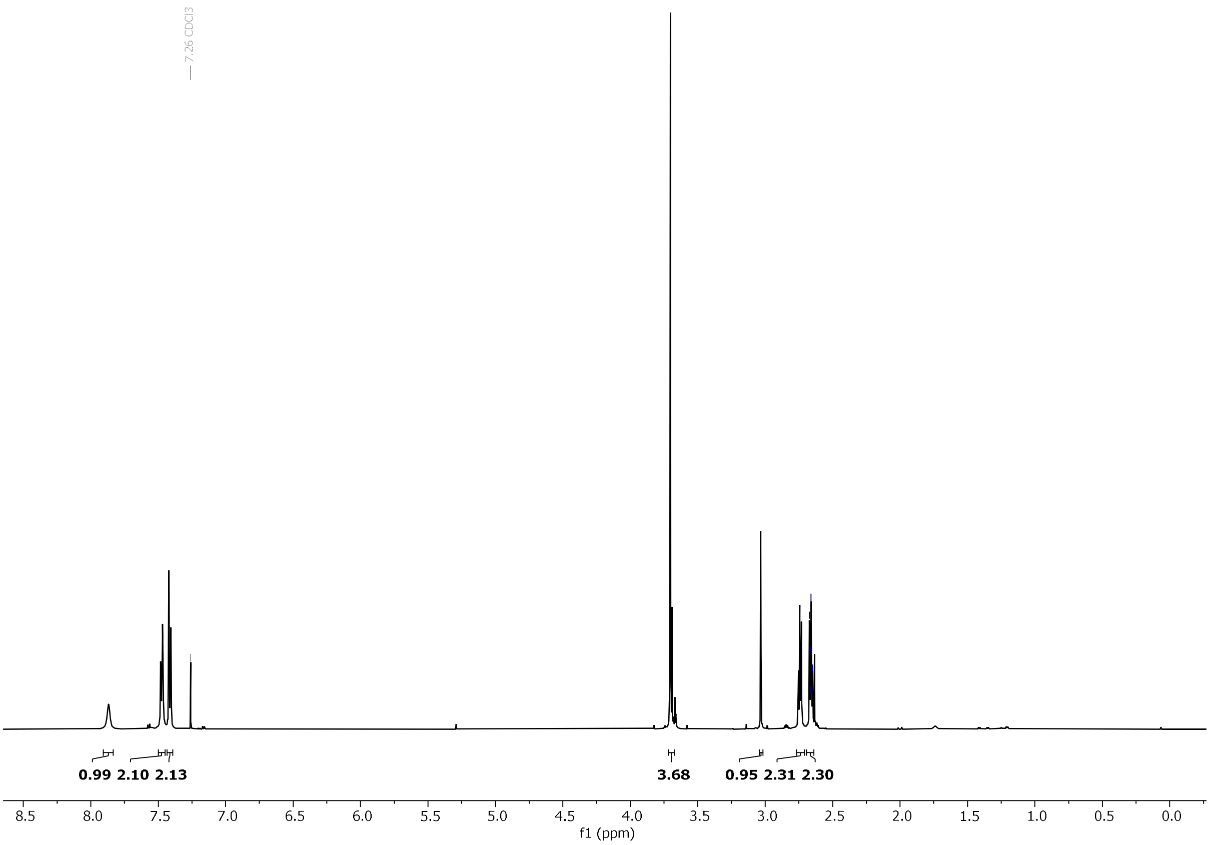


**Figure S1.** $^{1}$H-NMR (400 MHz, $CDCl_3$) of **(2)**.

**DADS(I)**

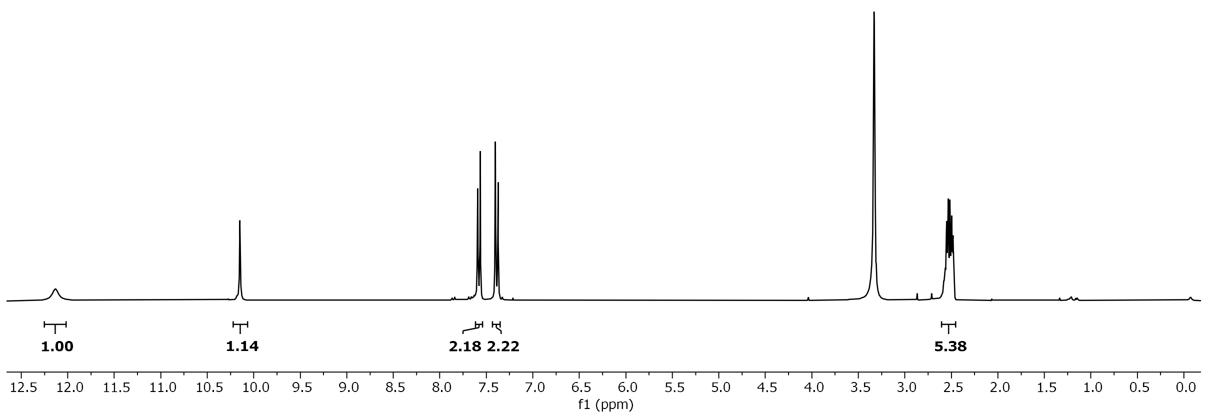


**Figure S2.** $^{1}$H-NMR (400 MHz, DMSO-d6) of DADS(I).

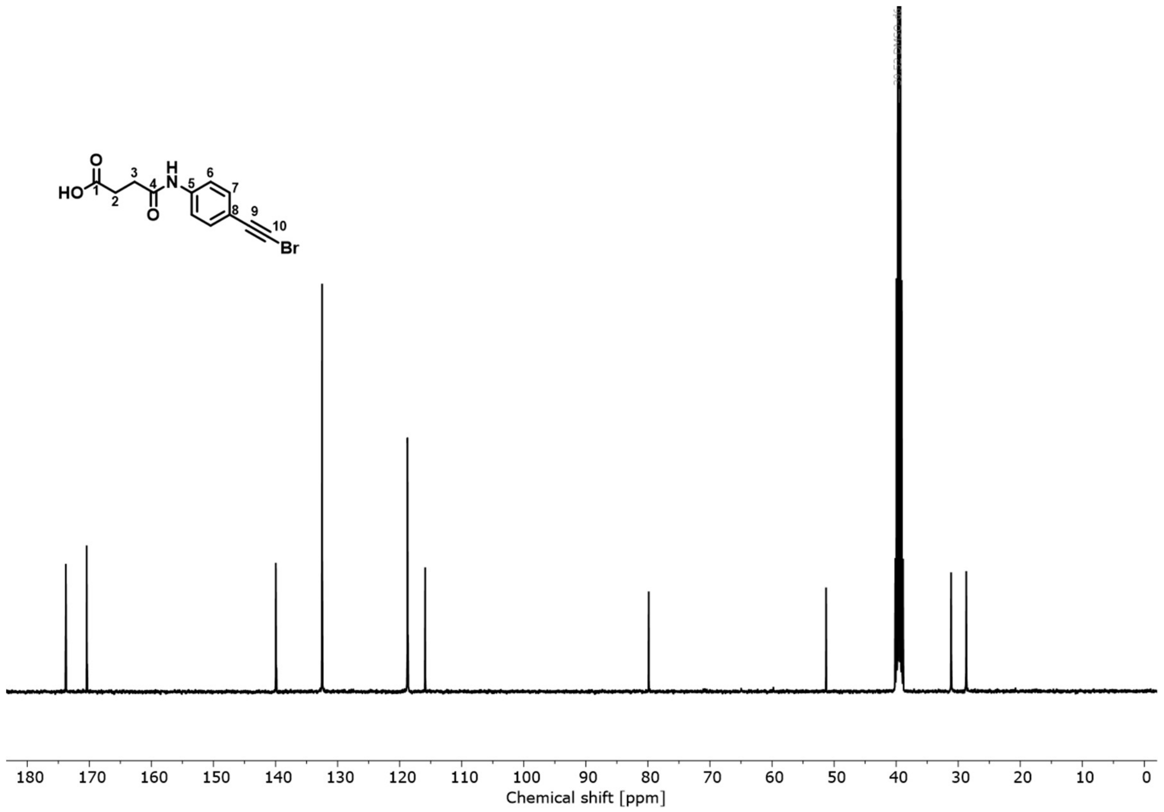


**Figure S3.** $^{13}$C-NMR (400 MHz, DMSO-d6) of DADS(I).

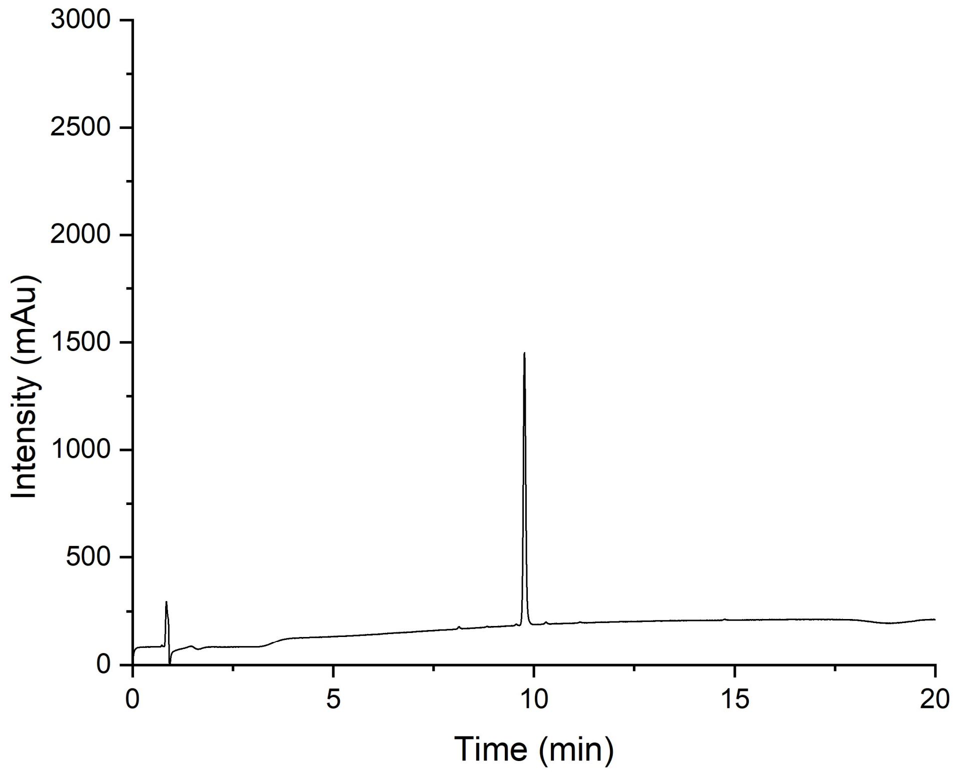


**Figure S4.** RP-HPLC chromatogram (from 95/5 to 5/95 Vol. % Water/acetonitrile with 0,1% formic acid in 20 min at 25 °C) of DADS(I).

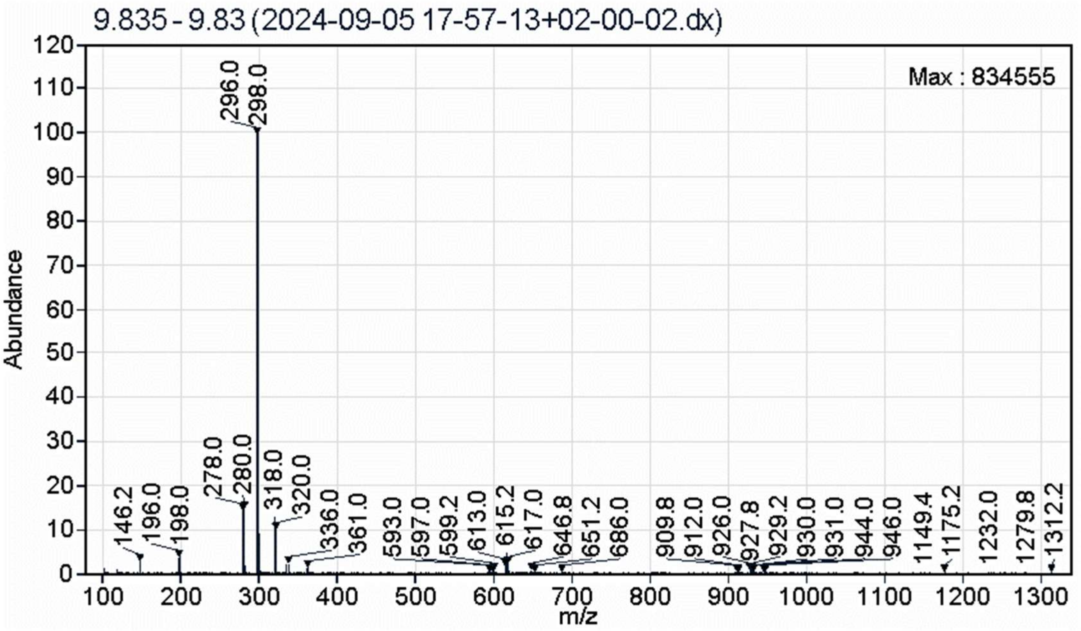


**Figure S5.** ESI-MS Spectrum of DADS(I).

**DADS(II)**

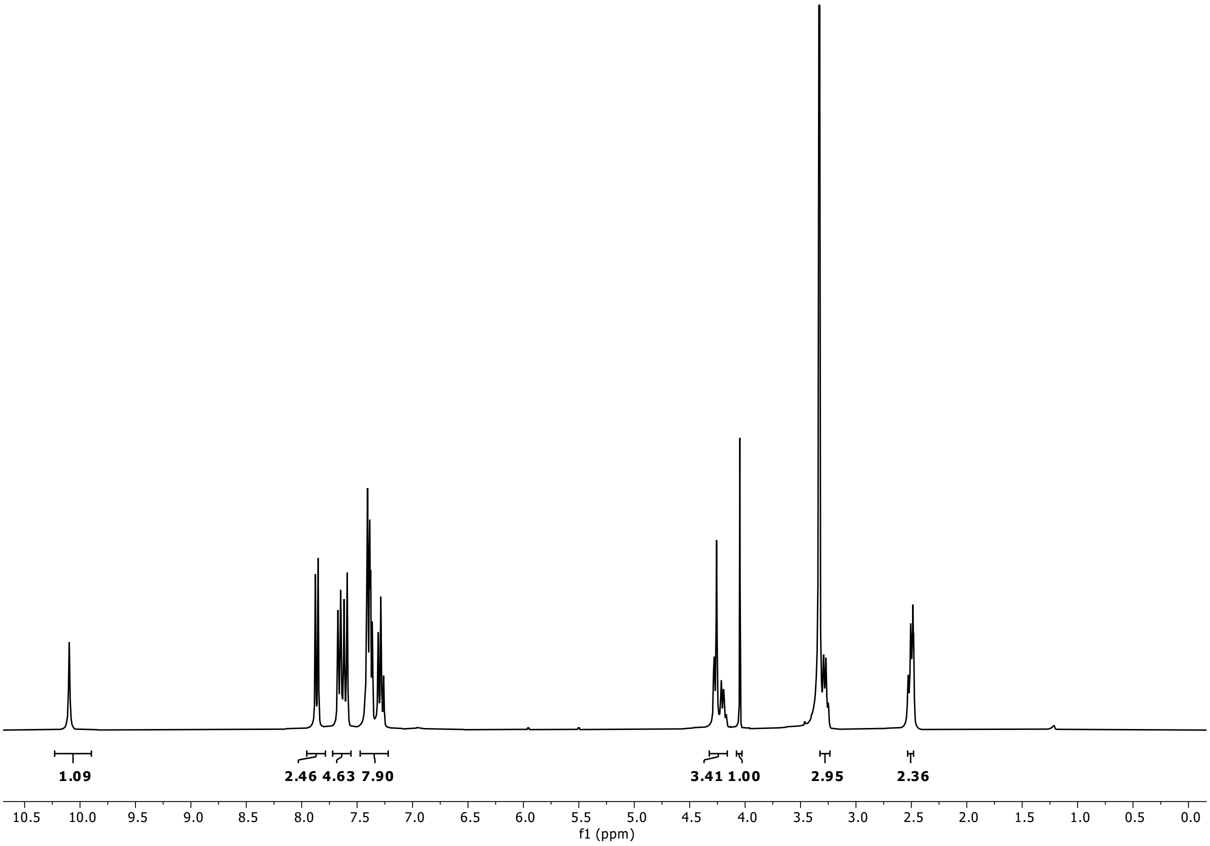


**Figure S6.** $^{1}$H-NMR (400 MHz, DMSO-d6) of DADS(II).

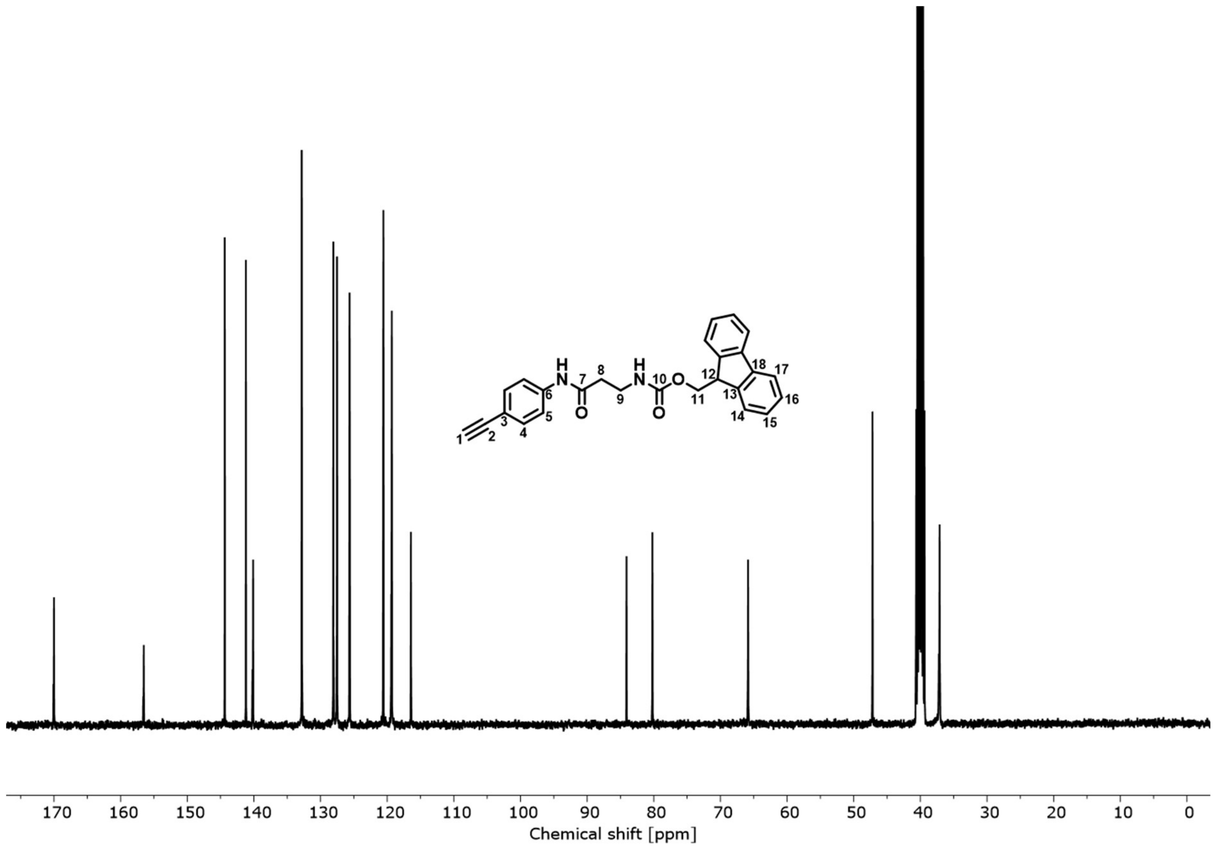


**Figure S7.** $^{13}$C-NMR (400 MHz, DMSO-d6) of DADS(II).

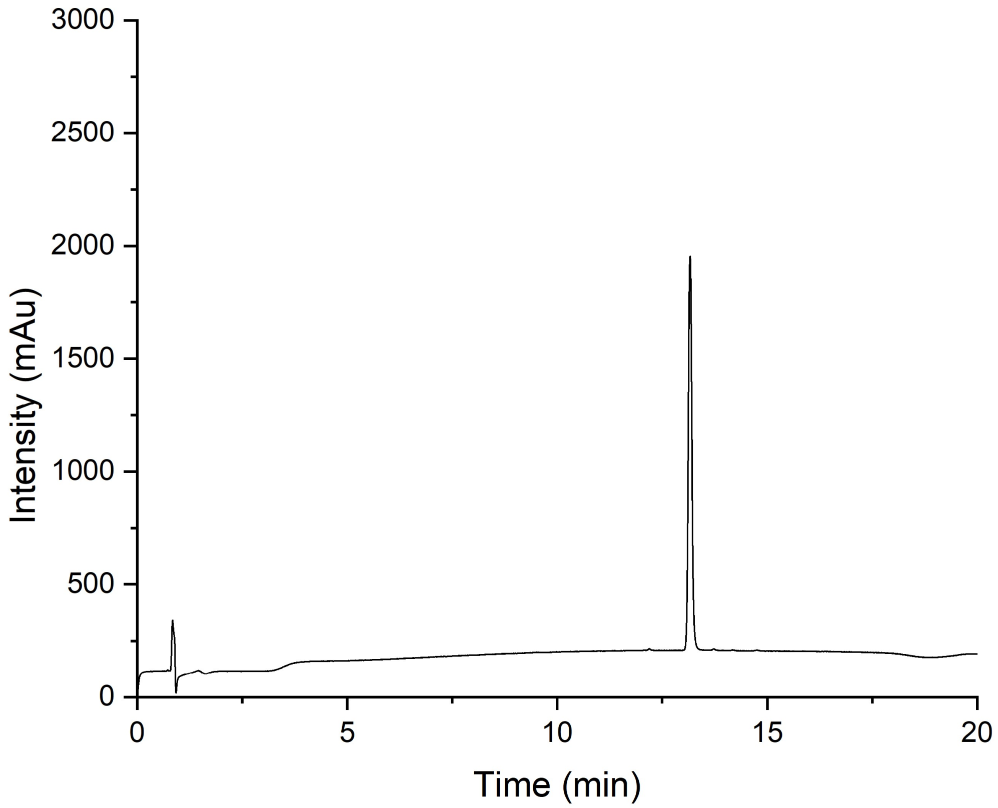


**Figure S8.** RP-HPLC chromatogram (from 95/5 to 5/95 Vol. % Water/acetonitrile with 0,1% formic acid in 20 min at 25 °C) of DADS(II).

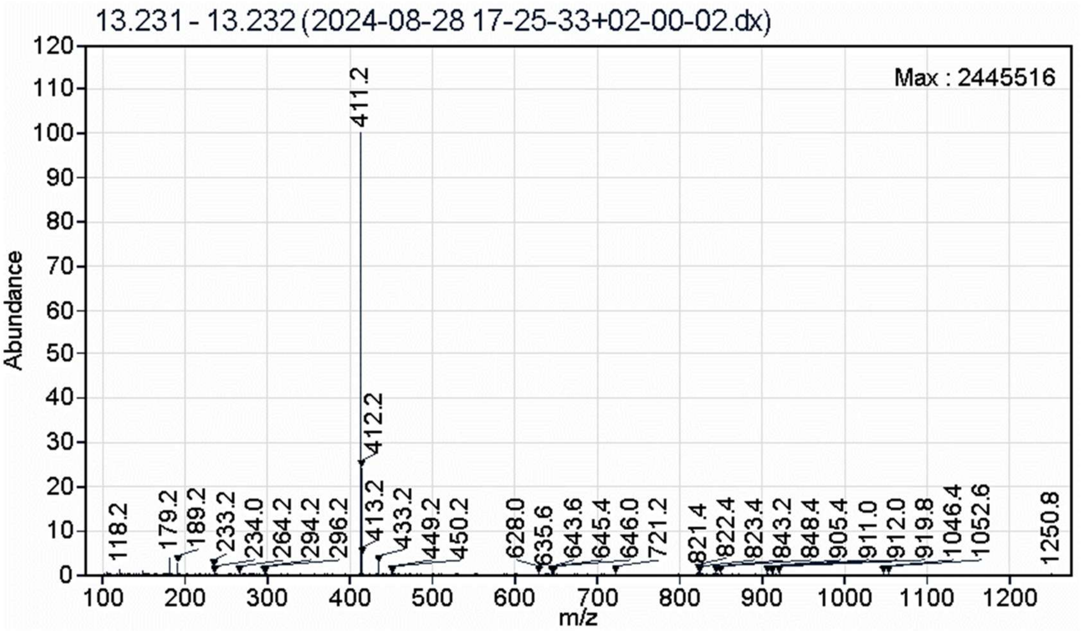


**Figure S9.** ESI-MS Spectrum of DADS(II).

**Test sequences for on-resin DADS synthesis and compatibility with SPPoS**

To investigate the assembly of diacetylene-containing macromolecular backbones via SPPoS, test sequences were synthesized. TentaGel S RAM resin, preloaded with EDS, was deprotected and subsequently conjugated with DADS (I) (for reaction conditions, see *General coupling protocol* and *Fmoc deprotection*). A **microcleavage** was then performed to assess the conversion by RP-HPLC-MS. The expected product was detected at a retention time of 8.66 min with a relative purity of 52% (**Figure S11**). In addition, a side product was observed at a retention time of 7.50 min, which could not be identified (**Figure S12**).

**1) EDS-DADS(I)**

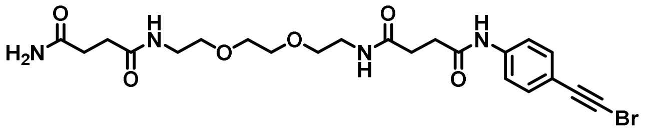


**RP-HPLC**: $t_R$ = 8.66 min, 52 % relative purity (UV), from 95/5 to 5/95 Vol. % Water/acetonitrile with 0,1% formic acid in 20 min at 25 °C.

**ESI-MS** calc. for C12H10BrNO3: $[M+1H]^{1+}$ 525.1; found 525.2 $[M+1H]^{1+}$.

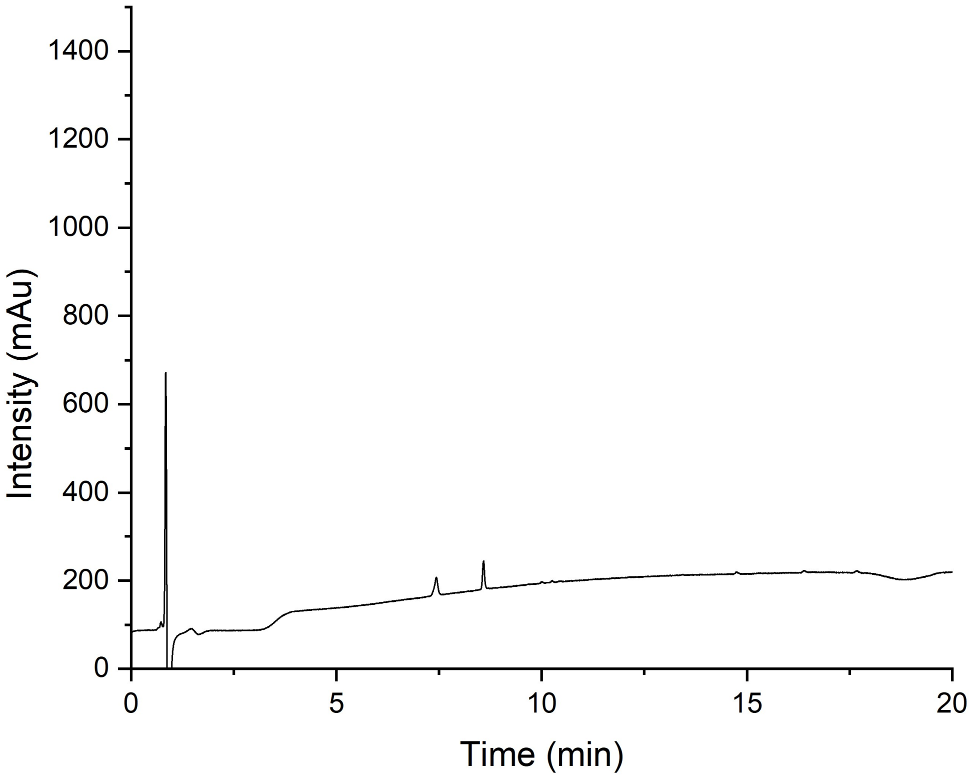


**Figure S10.** RP-HPLC chromatogram (from 95/5 to 5/95 Vol. % Water/acetonitrile with 0,1% formic acid in 20 min at 25 °C) of EDS-DADS(I) ($t_R$ = 8.66) and an unknown side product ($t_R$ = 7.50).

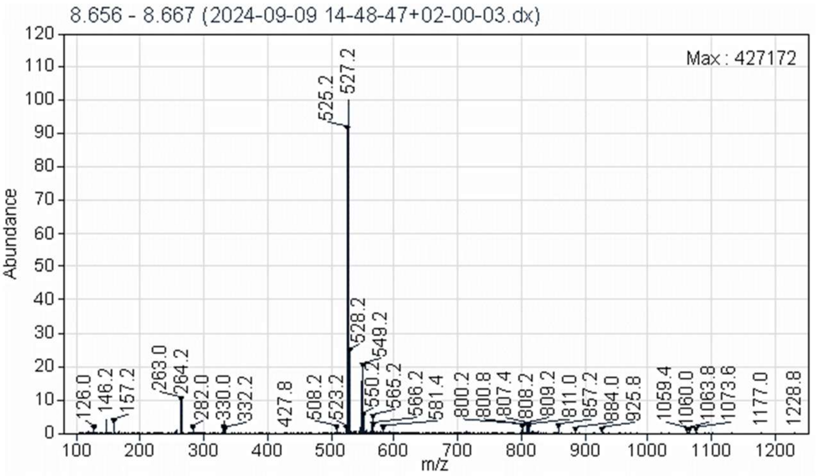


**Figure S11.** ESI-MS Spectrum of EDS-DADS(I) ($t_R$ = 8.66).

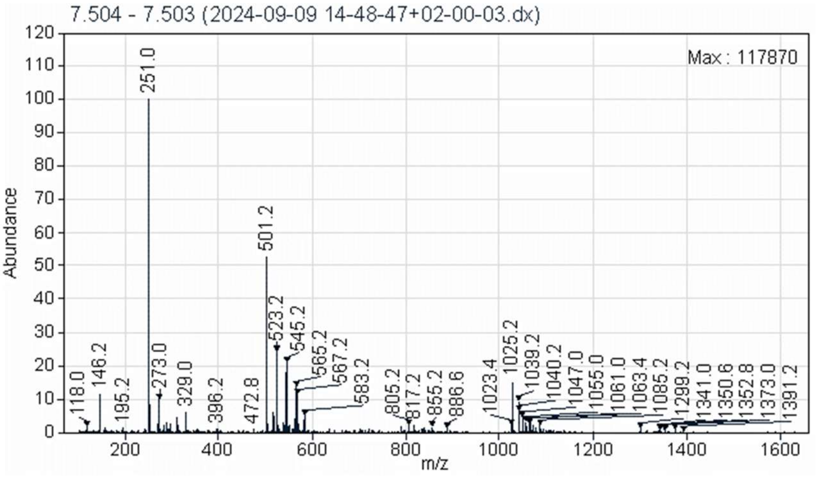


**Figure S12.** ESI-MS Spectrum of the unknown side product ($t_R$ = 7.50).

Subsequently, DADS (II) was conjugated to the resin-bound oligomer EDS–DADS (I) via a Cadiot–Chodkiewicz coupling (for reaction conditions, see *On-resin Cadiot–Chodkiewicz Coupling*). A microcleavage was then performed to assess the conversion by RP-HPLC-MS. The expected product (EDS–DADS–Fmoc) was identified at a retention time of 12.17 min with a relative purity of 90% (**Figure S14**). In addition, two side products were detected: one corresponding to a homodimerization product (side product (1), **Figure S15**) and the other to the product lacking the Fmoc group (side product (2), **Figure S16**). The unidentified side product from the previous conjugation step could no longer be detected and is therefore considered negligible for the subsequent coupling steps.

## 2) EDS-DADS-Fmoc

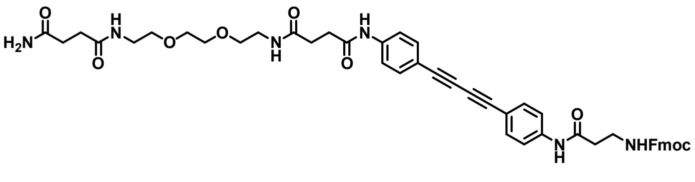

**RP-HPLC**: $t_R$ = 12.17 min, 90 % relative purity (UV), from 95/5 to 5/95 Vol. % Water/acetonitrile with 0,1% formic acid in 20 min at 25 °C.

**ESI-MS** calc. for C48H50N6O9: $[M+1H]^{1+}$ 855.4, $[M+2H]^{2+}$ 428.2; found 855.6 $[M+1H]^{1+}$, 428.4 $[M+2H]^{2+}$.

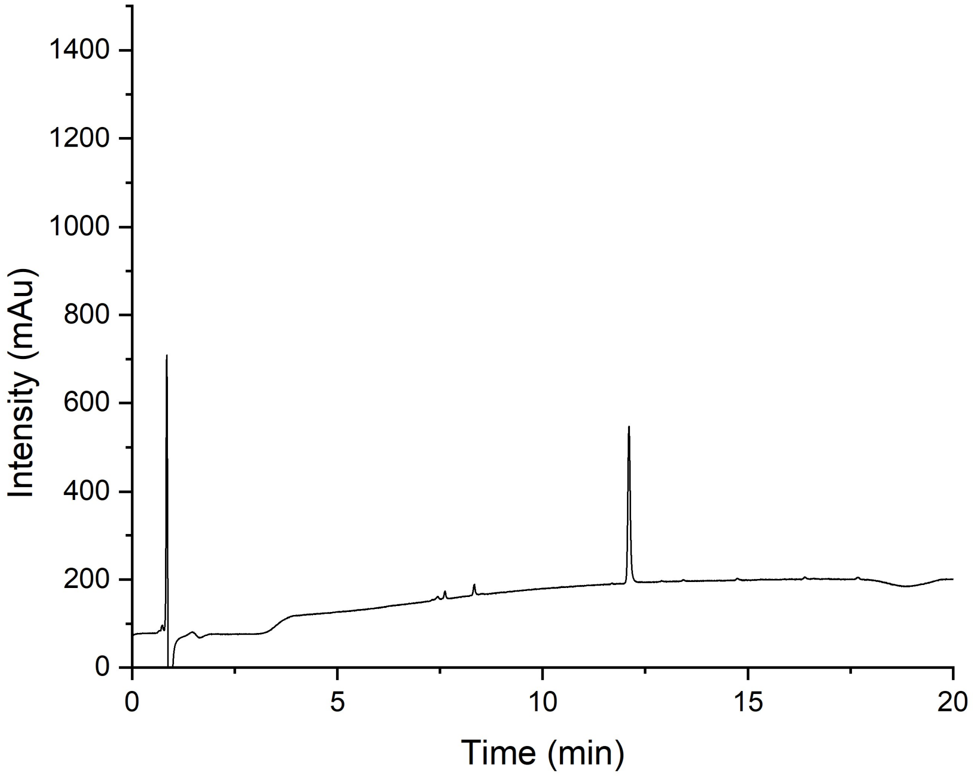


**Figure S13.** RP-HPLC chromatogram (from 95/5 to 5/95 Vol. % Water/acetonitrile with 0,1% formic acid in 20 min at 25 °C) of EDS-DADS-Fmoc.

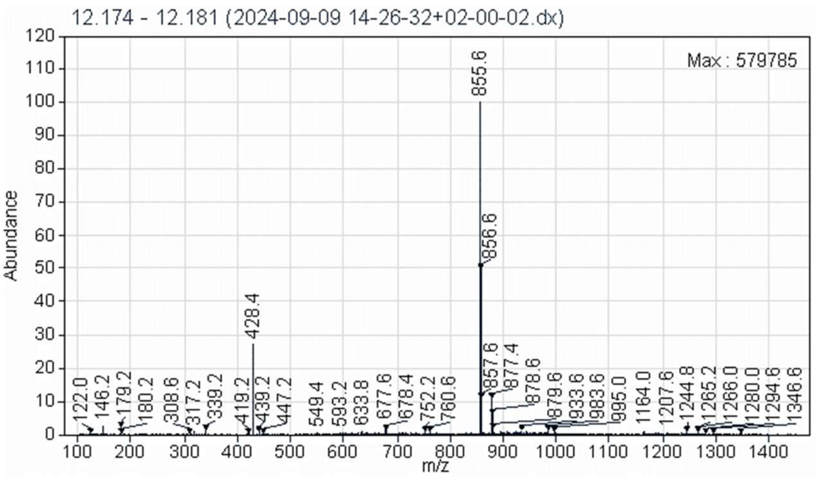


**Figure S14.** ESI-MS Spectrum of EDS-DADS-Fmoc ($t_R$ = 12.17).

Side product (1):

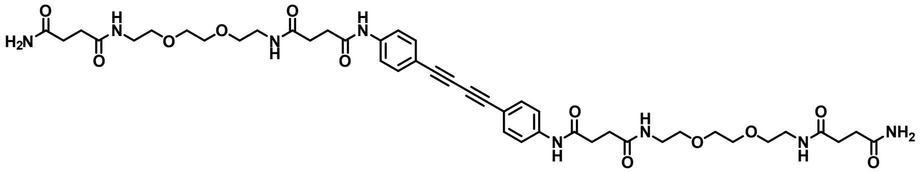

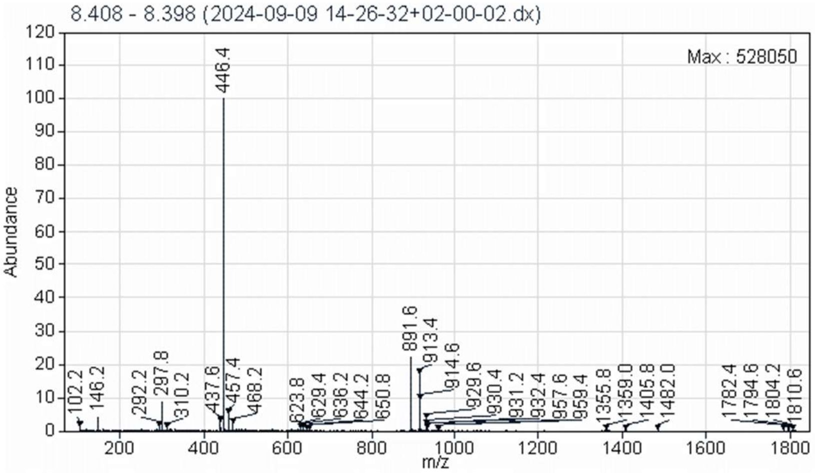


**Figure S15.** ESI-MS Spectrum of side product (1) ($t_R$ = 8.41).

Side product (2):

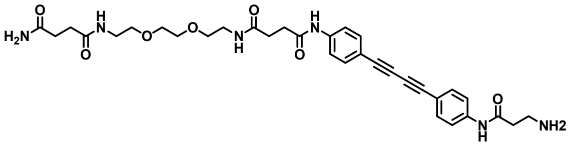

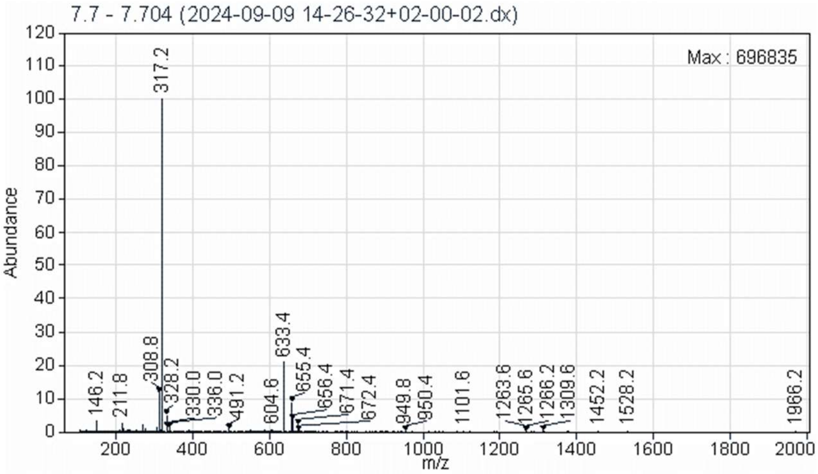


**Figure S16.** ESI-MS Spectrum of side product (2) ($t_R$ = 7.70).

Next, the terminal Fmoc protecting group was removed, and EDS was conjugated under standard conditions. The conversion was evaluated by microcleavage followed by RP-HPLC-MS analysis. The expected product (EDS–DADS–EDS–Fmoc) was identified at a retention time of 10.89 min with a relative purity of >98% (**Figure S18**). Side products detected in the previous analyses could not be observed and are therefore attributed to side reactions occurring under the microcleavage conditions, but are negligible for the backbone assembly on the solid phase.

### 3) EDS-DADS-EDS-Fmoc

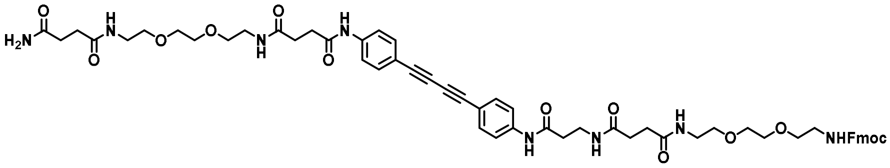

**RP-HPLC**: $t_R$ = 10.89 min, >98 % relative purity (UV), from 95/5 to 5/95 Vol. % Water/acetonitrile with 0,1% formic acid in 20 min at 25 °C.

**ESI-MS** calc. for $C_{58}H_{68}N_8O_{13}$: $[M+1H]^{1+}$ 1085.5, $[M+2H]^{2+}$ 543.3; found 1085.6 $[M+1H]^{1+}$, 543.6 $[M+2H]^{2+}$.

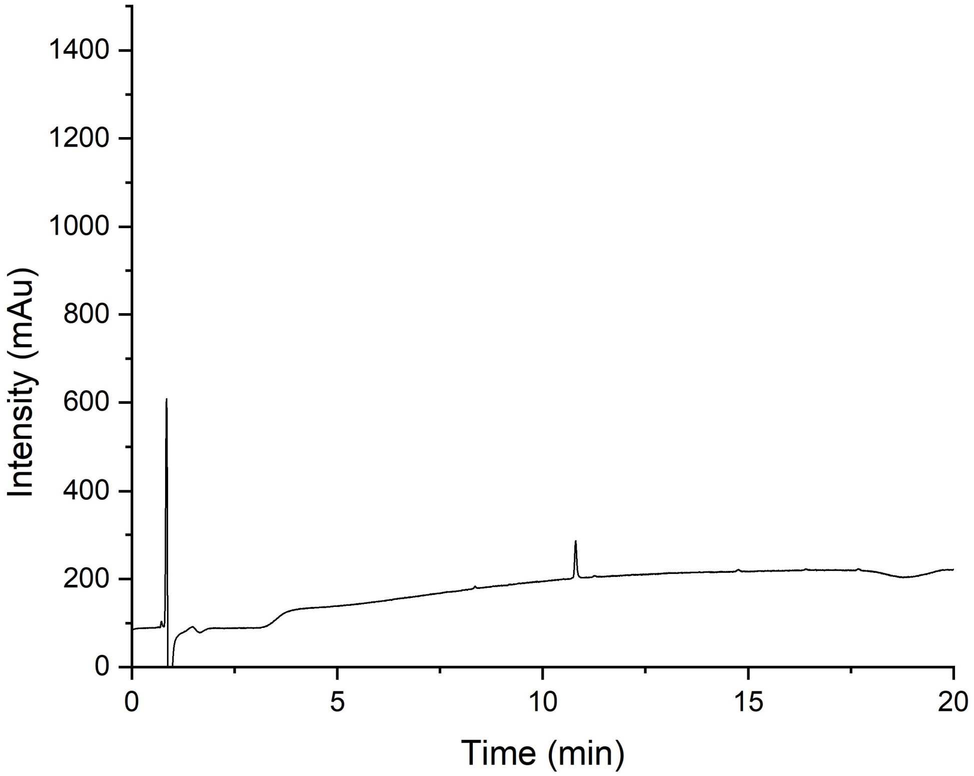

**Figure S17.** RP-HPLC chromatogram (from 95/5 to 5/95 Vol. % Water/acetonitrile with 0,1% formic acid in 20 min at 25 °C) of EDS-DADS-EDS-Fmoc.

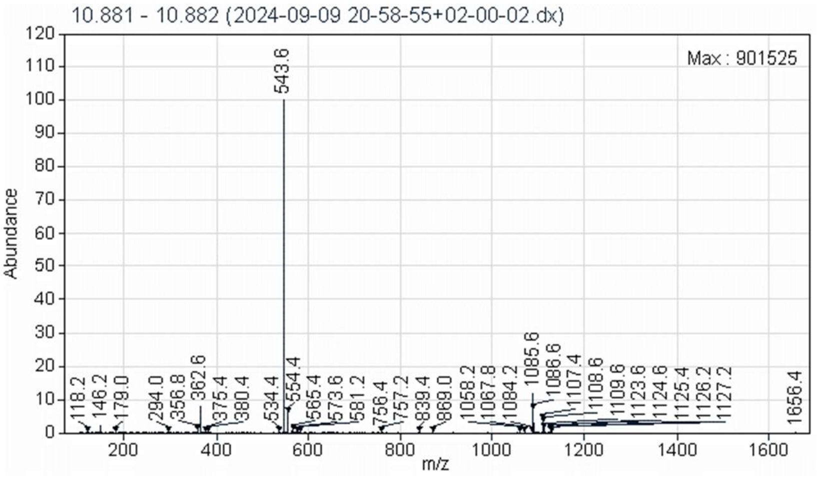


**Figure S18.** ESI-MS spectrum of EDS-DADS-EDS-Fmoc.

To investigate the orthogonality of the diacetylene functionality with respect to CuAAC, 4-pentynoic acid was subsequently conjugated *N*-terminally after prior Fmoc deprotection under standard conditions. The conversion was again evaluated by microcleavage followed by RP-HPLC-MS analysis. The expected product (EDS–DADS–EDS–PA) was identified at a retention time of 8.85 min with a relative purity of >95% (**Figure S20**).

4) EDS-DADS-EDS-PA

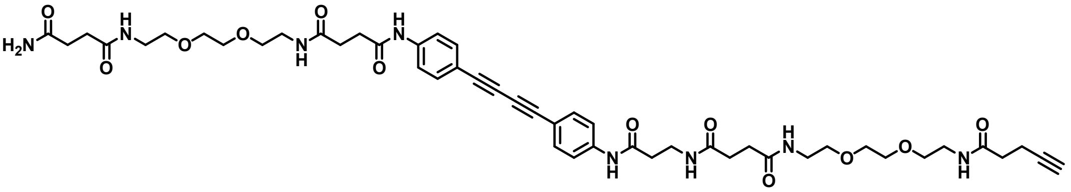

**RP-HPLC**: $t_R$ = 8.85 min, >95 % relative purity (UV), from 95/5 to 5/95 Vol. % Water/acetonitrile with 0,1% formic acid in 20 min at 25 °C.

**ESI-MS** calc. for C48H62N8O12: $[M+1H]^{1+}$ 943.5, $[M+2H]^{2+}$ 472.3; found 943.6 $[M+1H]^{1+}$, 472.4 $[M+2H]^{2+}$.

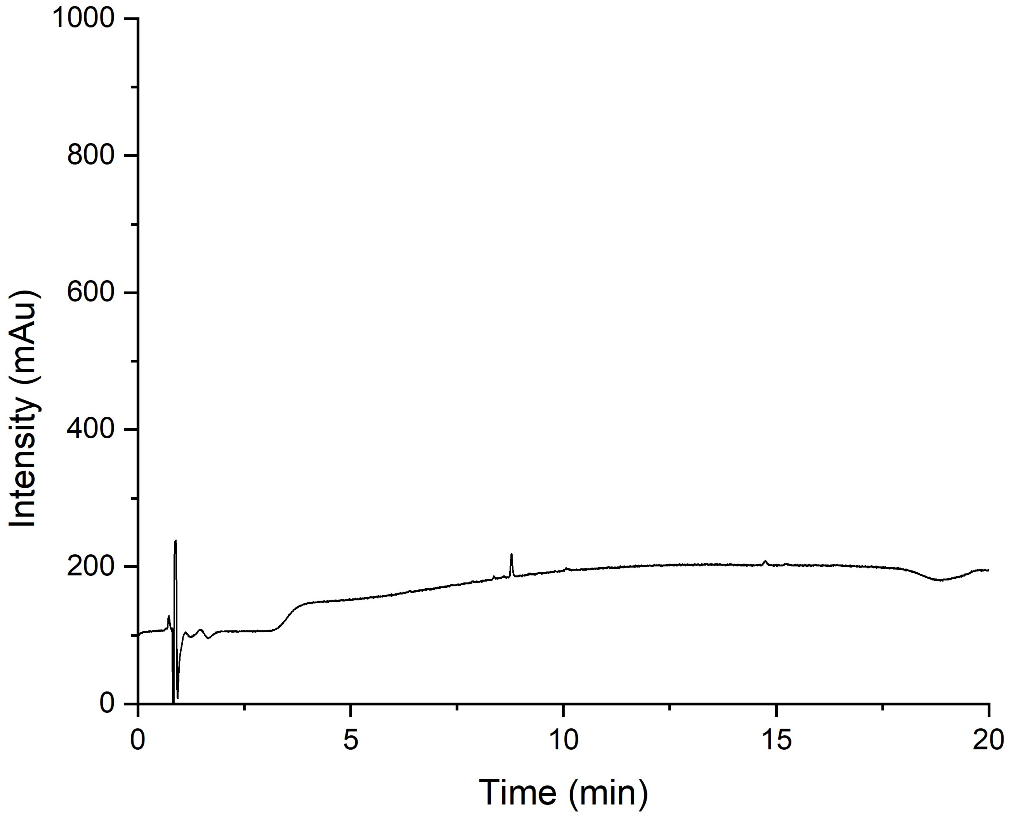


**Figure S19.** RP-HPLC chromatogram (from 95/5 to 5/95 Vol. % Water/acetonitrile with 0,1% formic acid in 20 min at 25 °C) of EDS-DADS-EDS-PA.

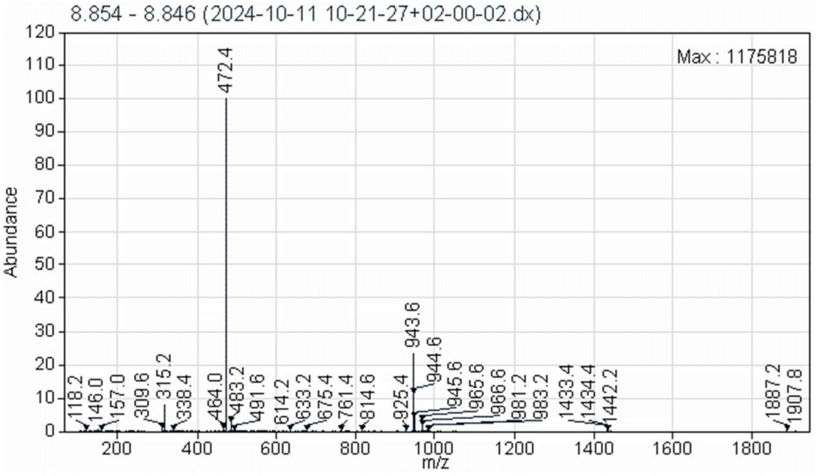


**Figure S20.** ESI-MS spectrum of EDS-DADS-EDS-PA.

Subsequently, the structure was glycosylated via CuAAC with peracetylated, azide-functionalized mannose (for reaction conditions, see CuAAC protocol for glycosylation). The expected product (EDS–DADS–EDS–PA–Man) was identified at a retention time of 9.33 min with a relative purity of 90% (**Figure S22**). Side products of lower mass are attributed to partial cleavage of the acetyl protecting groups. Additionally, a side product was detected at $t_R$ = 7.99 min, in which the diacetylene unit had been cleaved and the mannose had been attached via CuAAC to the terminal alkyne (**Figure S23**, side product). These results indicate that the diacetylene unit is partially labile under the CuAAC conditions, and glycosylation of glyco-functionalized diacetylene-containing macromolecules should therefore be performed prior to the formation of the diacetylene backbone via Cadiot–Chodkiewicz coupling.

**5) EDS-DADS-EDS-PA-Man**

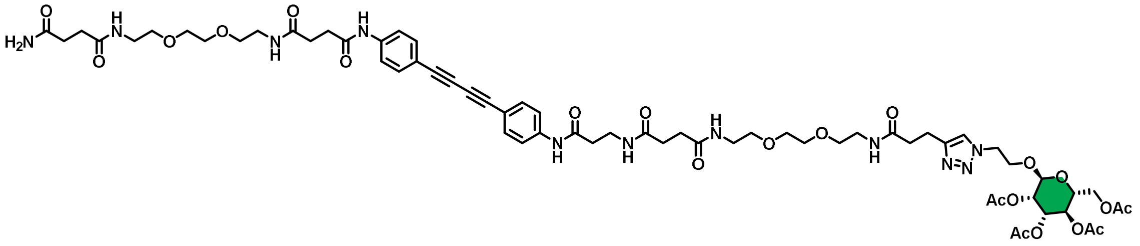


**RP-HPLC**: $t_R$ = 9.33 min, 90 % relative purity (UV), from 95/5 to 5/95 Vol. % Water/acetonitrile with 0,1% formic acid in 20 min at 25 °C.

**ESI-MS** calc. for C48H62N8O12: $[M+2H]^{2+}$ 680.8, $[M+3H]^{3+}$ 454.2; found 681.0 $[M+2H]^{2+}$, $[M+3H]^{3+}$454.4.

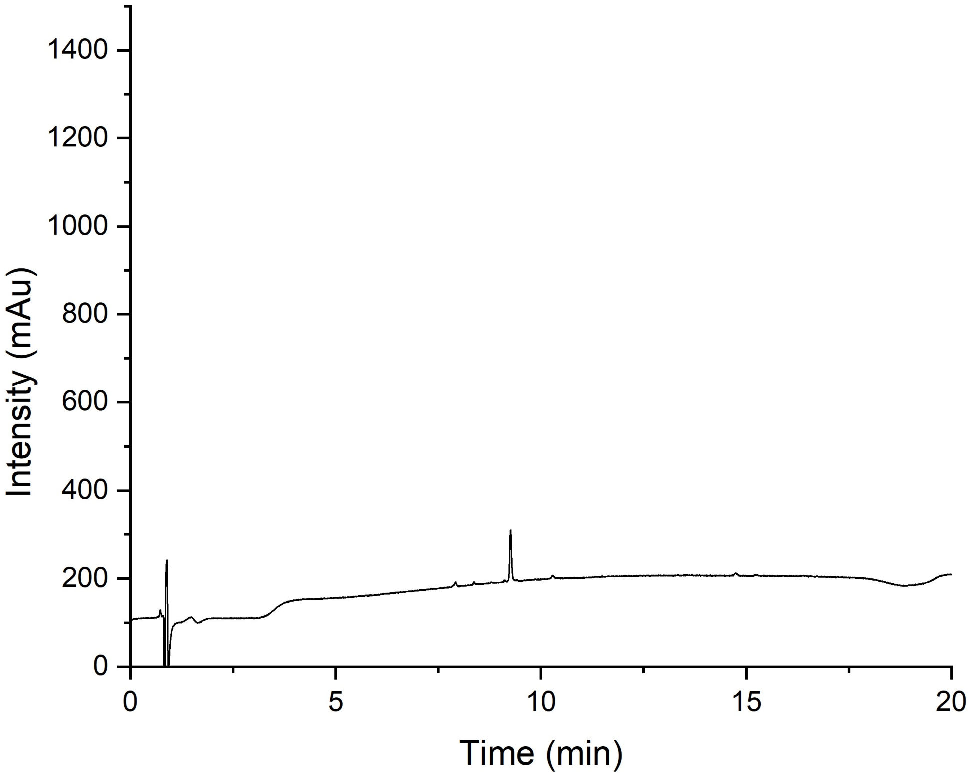


**Figure S21.** RP-HPLC chromatogram (from 95/5 to 5/95 Vol. % Water/acetonitrile with 0,1% formic acid in 20 min at 25 °C) of EDS-DADS-EDS-PA-Man.

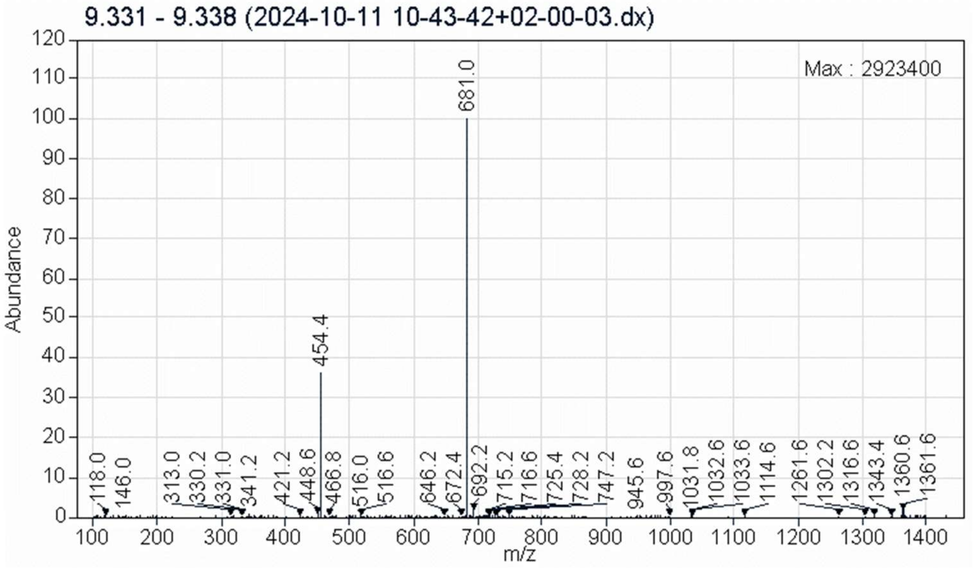


**Figure S22.** ESI-MS spectrum of EDS-DADS-EDS-PA-Man ($t_R$ = 9.33).

Side product

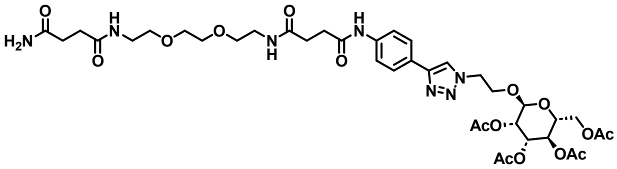


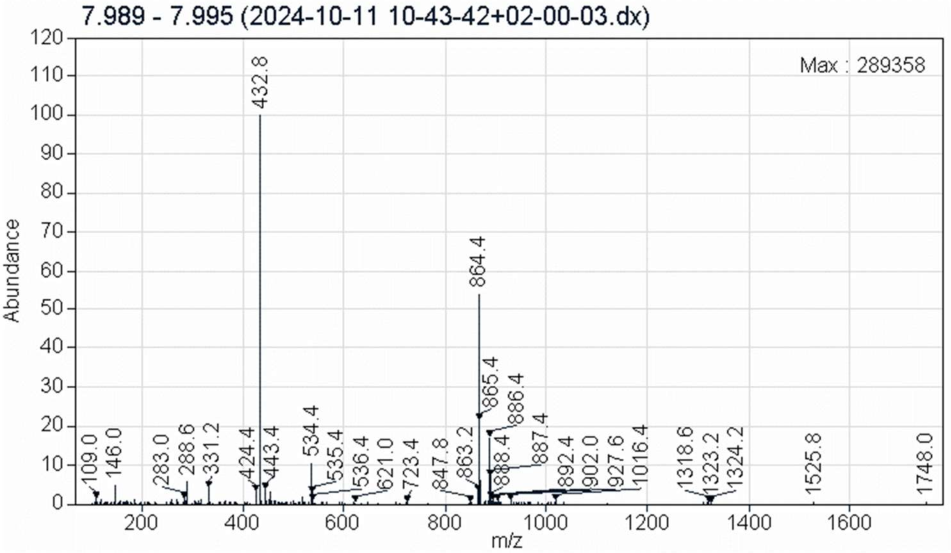


**Figure S23.** ESI-MS spectrum of the Side product ($t_R$ = 7.99).

**Glycooligomer Man$_4$DA-Chol**

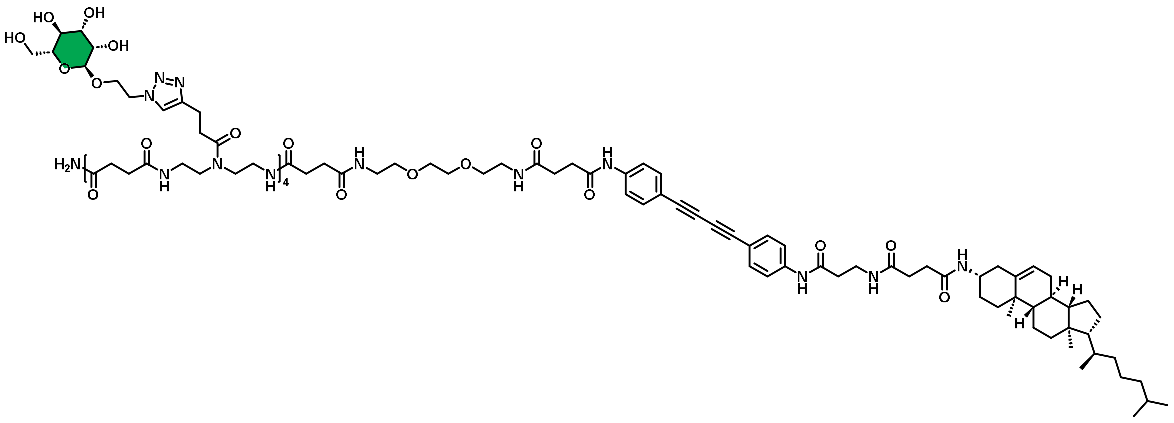

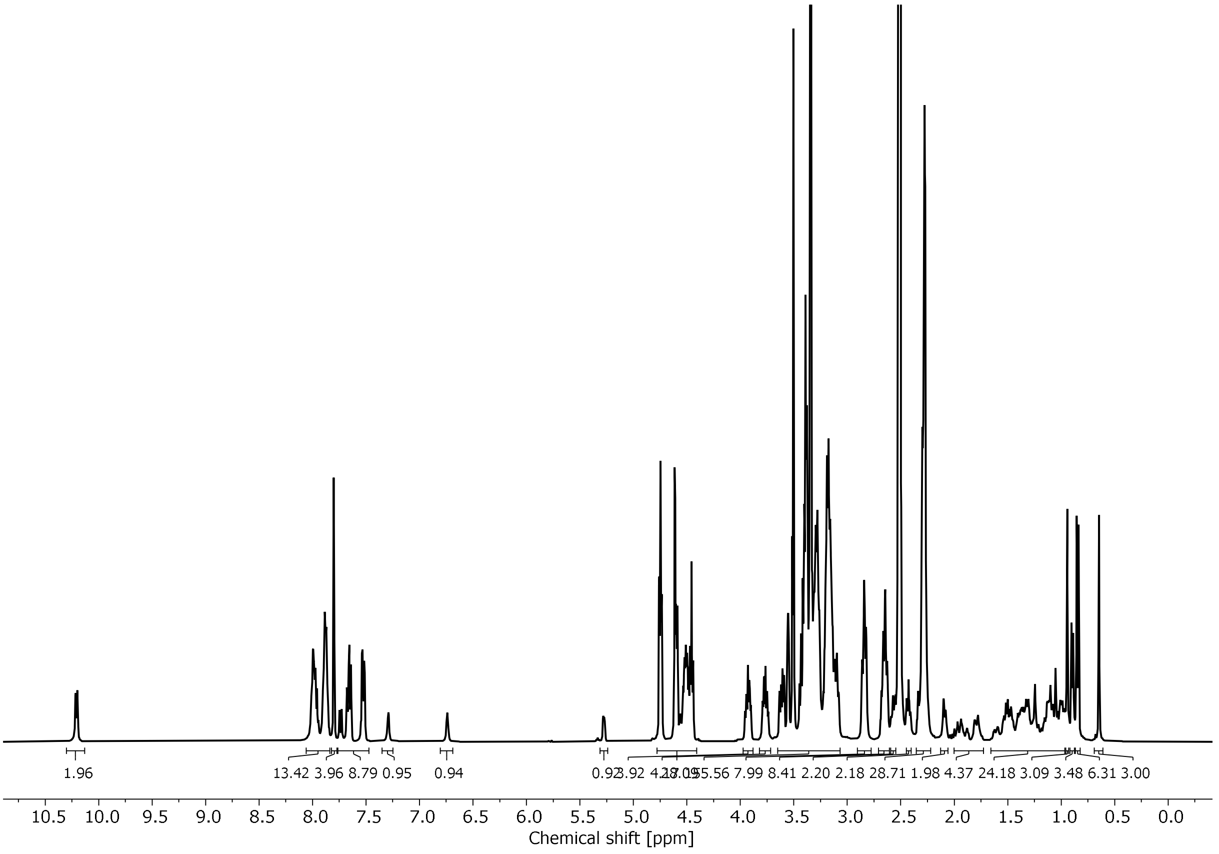


**Figure S24.** $^1$H-NMR (400 MHz, DMSO-d6) of Man$_4$DA-Chol.

**$^1$H-NMR (400 MHz, DMSO-d6) δ [ppm]** = 10.21 (d, *J* = 6.8 Hz, 2H, Ar-N*H*), 8.04 – 7.84 (m, 11H, Amide-N*H*), 7.82 – 7.78 (m, 4H, Triazole-C*H*), 7.76 – 7.49 (m, 9H, Chol-N*H*, Ar-C*H*), 7.29 (s, 1H, C(O)N$\mathit{H_2}$), 6.74 (s, 1H, C(O)N$\mathit{H_2}$), 5.29 – 5.26 (m, 1H, Chol-C=C*H*), 4.78 – 4.43 (m, 28H, Man-O*H*, Anom.-C*H*, Triazole-NC$\mathit{H_2}$), 3.98 – 3.88 (m, 4H, Man-C*H*OH), 3.82 – 3.72 (m, 4H, Man-C*H*OH), 3.65 – 3.06 (m, 83H, Chol-NHC*H*, β-Ala-C$\mathit{H_2}$NH, DADS-NHC(O)CH$_2$C$\mathit{H_2}$NH, EDS-C$\mathit{H_2}$, TDS-C$\mathit{H_2}$NC$\mathit{H_2}$, TDS-C$\mathit{H_2}$NHC(O), Man-C*H*OH, Man-C*H*C$\mathit{H_2}$OH, Triazole-NCH$_2$C$\mathit{H_2}$O), 2.84 (m, 8H

TDS-NC(O)CH$_2$C$H_2$C), 2.71 – 2.61 (m, 8H,TDS-NC(O)C$H_2$CH$_2$C), 2.57 (t, J = 7.1 Hz, 2H, DADS-NHC(O)C$H_2$CH$_2$C(O)NH), 2.43 (t, *J* = 7.1 Hz, 2H, DADS-NHC(O)C$H_2$CH$_2$NH), 2.37 – 2.22 (m, 28H, C(O)C$H_2$C$H_2$C(O)), 2.12-2.05 (m, 2H, Chol-NHC(O)C$H_2$), 2.00 – 1.73 (m, 4H, Chol-C=CHC$H_2$, Chol-C=CC$H_2$), 1.66 – 0.97 (m, 24H, Chol-C*H*, Chol-C$H_2$), 0.96 – 0.82 (m, 12H, Chol-C$H_3$), 0.65 (s, 3H, Chol-C$H_3$).

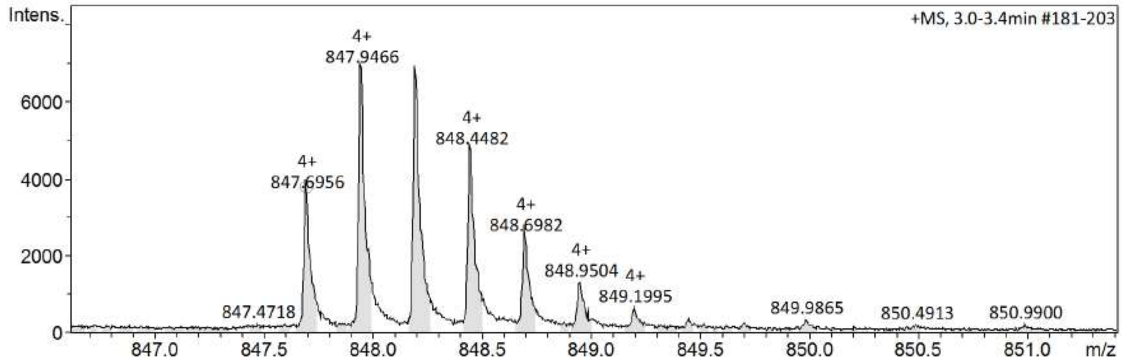


| Meas. m/z | # | Ion Formula | m/z | err [ppm] | mSigma | # mSigma | Score | rdb | e⁻ Conf | N-Rule |
|---|---|---|---|---|---|---|---|---|---|---|
| 847.6956 | 1 | C158H247N33O49 | 847.6957 | 0.2 | 22.9 | 1 | 100.00 | 52.0 | even | ok |
| | 2 | C153H247N35O51 | 847.6947 | -1.0 | 27.8 | 2 | 63.97 | 48.0 | even | ok |

**Figure S25.** HR-ESI-MS of Man$_4$DA-Chol.

**HR-ESI-MS:** for $C_{158}H_{243}N_{33}O_{49}$ m/z $[M+4H]^{4+}$ calcd.: 847.6954, found: 847.6956
mass error 0.235934 ppm.

**Glycooligomer Gal$_4$DAChol**

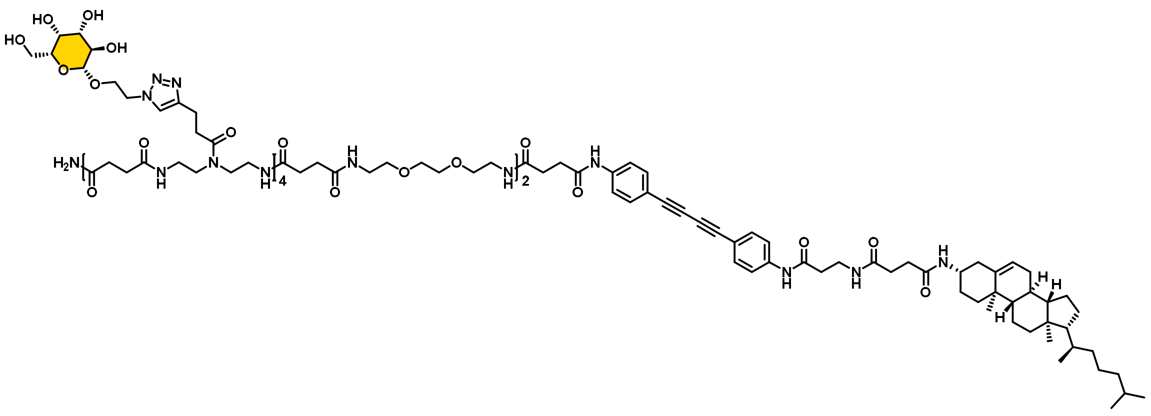

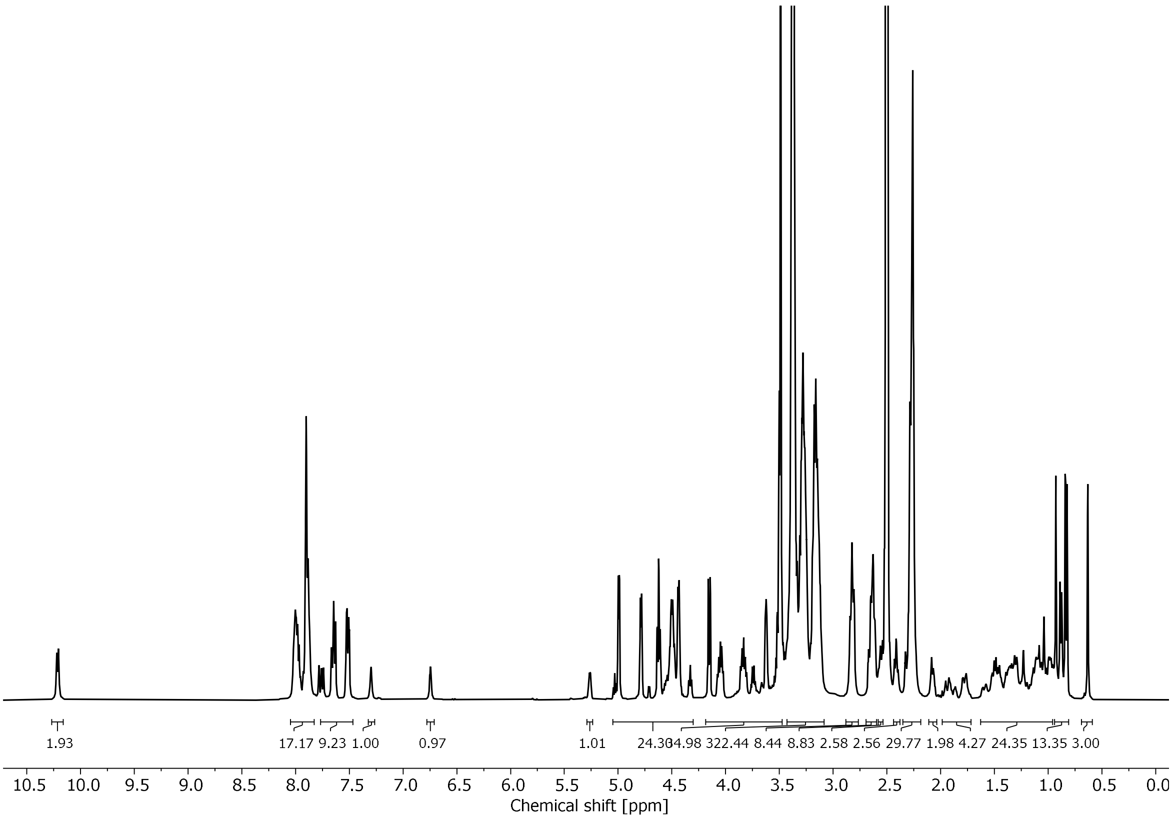


**Figure S28.** $^1$H-NMR (400 MHz, DMSO-d6) of Gal$_4$DA-Chol.

**$^1$H-NMR (400 MHz, DMSO-d6) δ [ppm] =** 10.21 (d, J = 6.4 Hz, 2H, Ar-N*H*), 8.05 – 7.81 (m, 15H, Amide-N*H,* Triazole-C*H*), 7.77 – 7.46 (m, 9H, Chol-N*H*, Ar-C*H*), 7.30 (s, 1H, C(O)N*H$_2$*), 6.75 (s, 1H, C(O)N*H$_2$*), 5.26 (d, J = 4.8 Hz, 1H, Chol-C=C*H*), 5.05 – 4.30 (m, 24H, Gal-O*H*, Triazole-NC*H$_2$*), 4.18 – 3.47 (m, 34H, Anom.-C*H,* Gal-C*H*OH, Gal-C*H*C*H$_2$*OH), 3.43 – 3.08 (m, 61H, Chol-NHC*H*, β-Ala-C*H$_2$*NH, DADS-NHC(O)CH$_2$*CH$_2$*NH, EDS-C*H$_2$*, TDS-C*H$_2$*NC*H$_2$,* TDS-C*H$_2$*NHC(O), Gal-C*H*OH, Triazole-NCH$_2$C*H$_2$*O), 2.88 – 2.76 (m, 8H, TDS-NC(O)CH$_2$C*H$_2$C*), 2.69 – 2.59 (m, 8H, TDS-NC(O)C*H$_2$*CH$_2$*C*), 2.57-2.53 (m, 2H, DADS-NHC(O)C*H$_2$*CH$_2$C(O)NH), 2.41 (t, J = 7.0 Hz, 2H, DADS-

NHC(O)C$H_2$CH$_2$NH), 2.35 – 2.18 (m, 28H, C(O)C$H_2$C$H_2$C(O)), 2.12-2.05 (m, 2H, Chol-NHC(O)C$H_2$), 1.98 – 1.72 (m, 4H, Chol-C=CHC$H_2$, Chol-C=CC$H_2$), 1.62 – 0.96 (m, 24H, Chol-C$H$, Chol-C$H_2$), 0.94 – 0.81 (m, 12H, Chol-C$H_3$), 0.63 (s, 3H, Chol-C$H_3$).

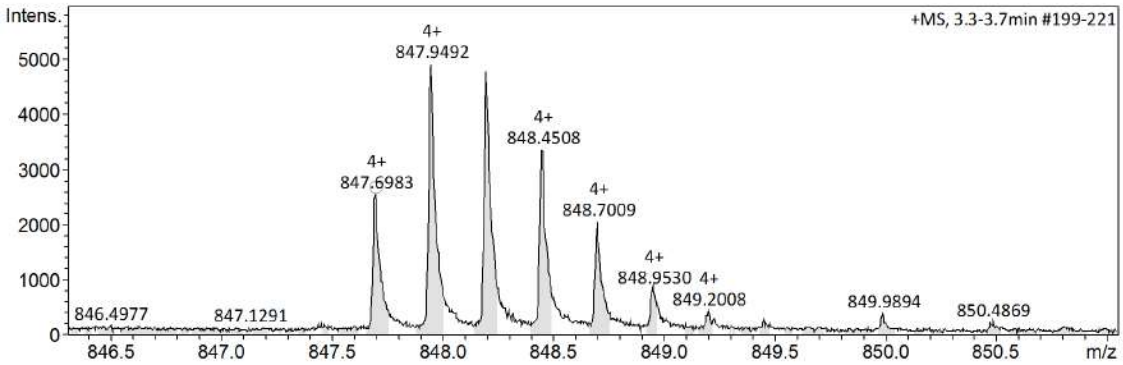


| Meas. m/z | # | Ion Formula | m/z | err [ppm] | mSigma | # mSigma | Score | rdb | e⁻ Conf | N-Rule |
|---|---|---|---|---|---|---|---|---|---|---|
| 847.6983 | 1 | C158H247N33O49 | 847.6957 | -3.1 | 25.1 | 1 | 21.88 | 52.0 | even | ok |
| | 2 | C157H247N35O48 | 847.6985 | 0.3 | 26.5 | 2 | 99.40 | 52.0 | even | ok |
| | 3 | C156H251N31O52 | 847.6982 | -0.1 | 28.0 | 3 | 100.00 | 47.0 | even | ok |
| | 4 | C155H251N33O51 | 847.7010 | 3.2 | 29.8 | 4 | 17.99 | 47.0 | even | ok |

**Figure S29.** HR-ESI-MS of Gal$_4$DA-Chol.

**HR-ESI-MS:** for $C_{158}H_{243}N_{33}O_{49}$ m/z $[M+4H]^{4+}$ calcd.: 847.6954, found: 847.6983
mass error 3.421040 ppm.

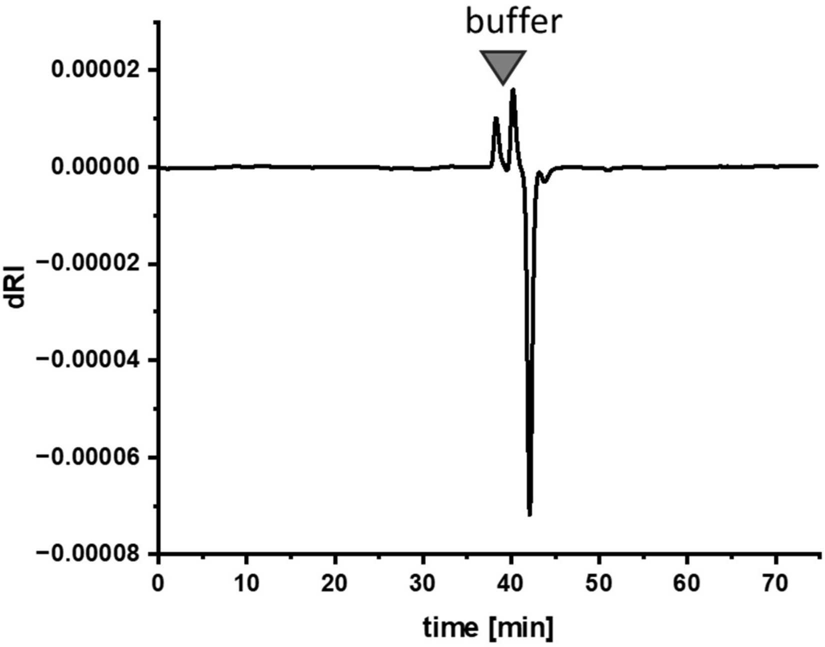


**Figure S30.** $H_2O$-SEC-MALS of non-irradiated $Man_4DA$-Chol.

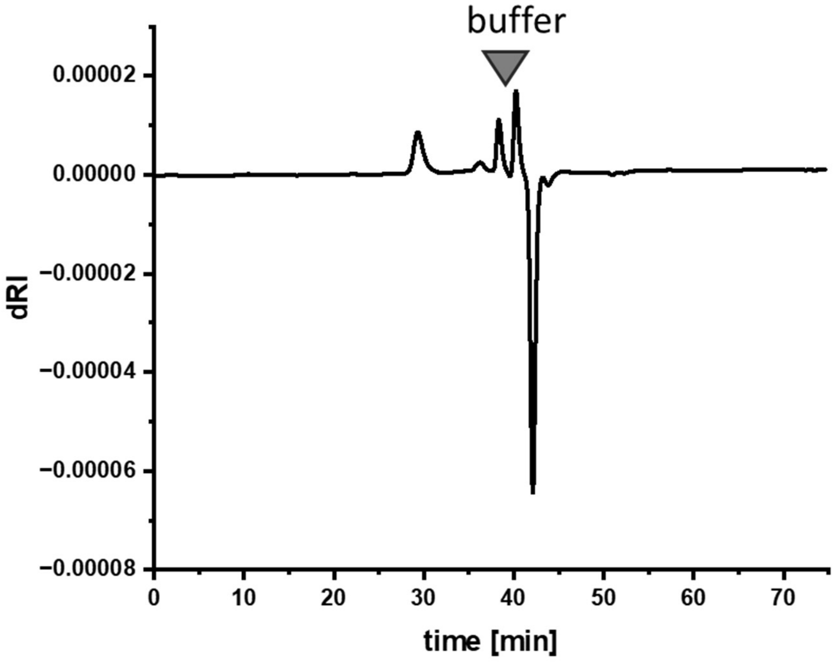


**Figure S31.** $H_2O$-SEC-MALS of irradiated $Man_4DA$-Chol.

Đ (via $H_2O$-SEC, MALS coupled RI-detector): 1.02

$M_n$ [kDa] (via $H_2O$-SEC, MALS coupled RI-detector): 354 (corresponding DP = 105)

Of note, a quantitative determination of the degree of polymerization (DP) based on GPC data is not meaningful in this case, since the amphiphilic diacetylene ligands form micelles in water and the irradiation-induced polymerization occurs within these aggregates. The resulting species are not individual polymer chains but rather crosslinked supramolecular assemblies, which do not elute in GPC according to their true molecular weight. Thus, the GPC traces can only be interpreted qualitatively to confirm that topochemical polymerization has occurred, but not to derive absolute values such as the degree of polymerization.

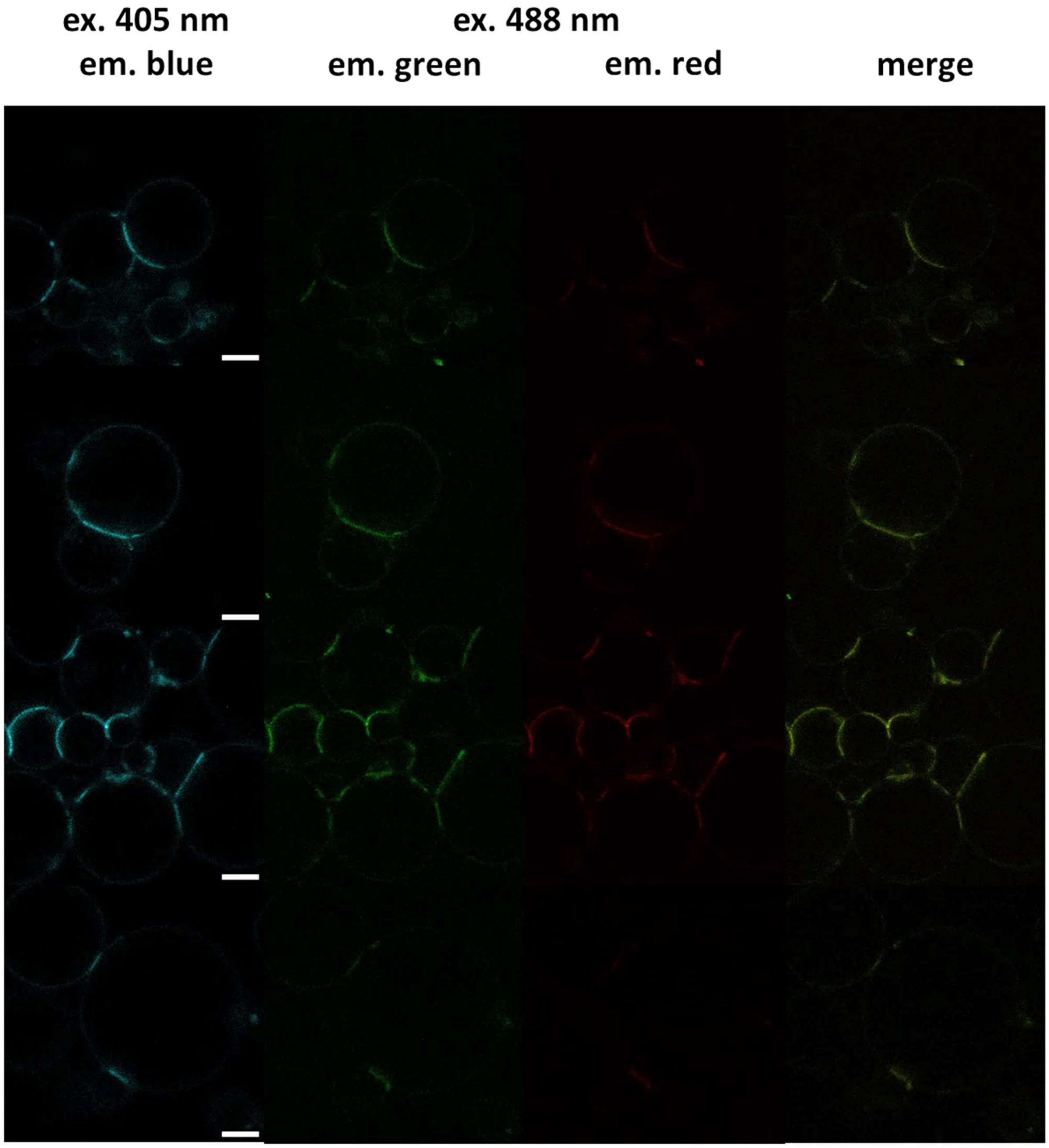


**Figure S32.** Additional fluorescence microscopy images of lectin clustered GUVs presenting Man$_4$DA-Chol before UV irradiation. Brightness adjusted for better visibility; high noise level in brightness adjusted images indicates low fluorescence / autofluorescence. Scale bars: 10 µm.

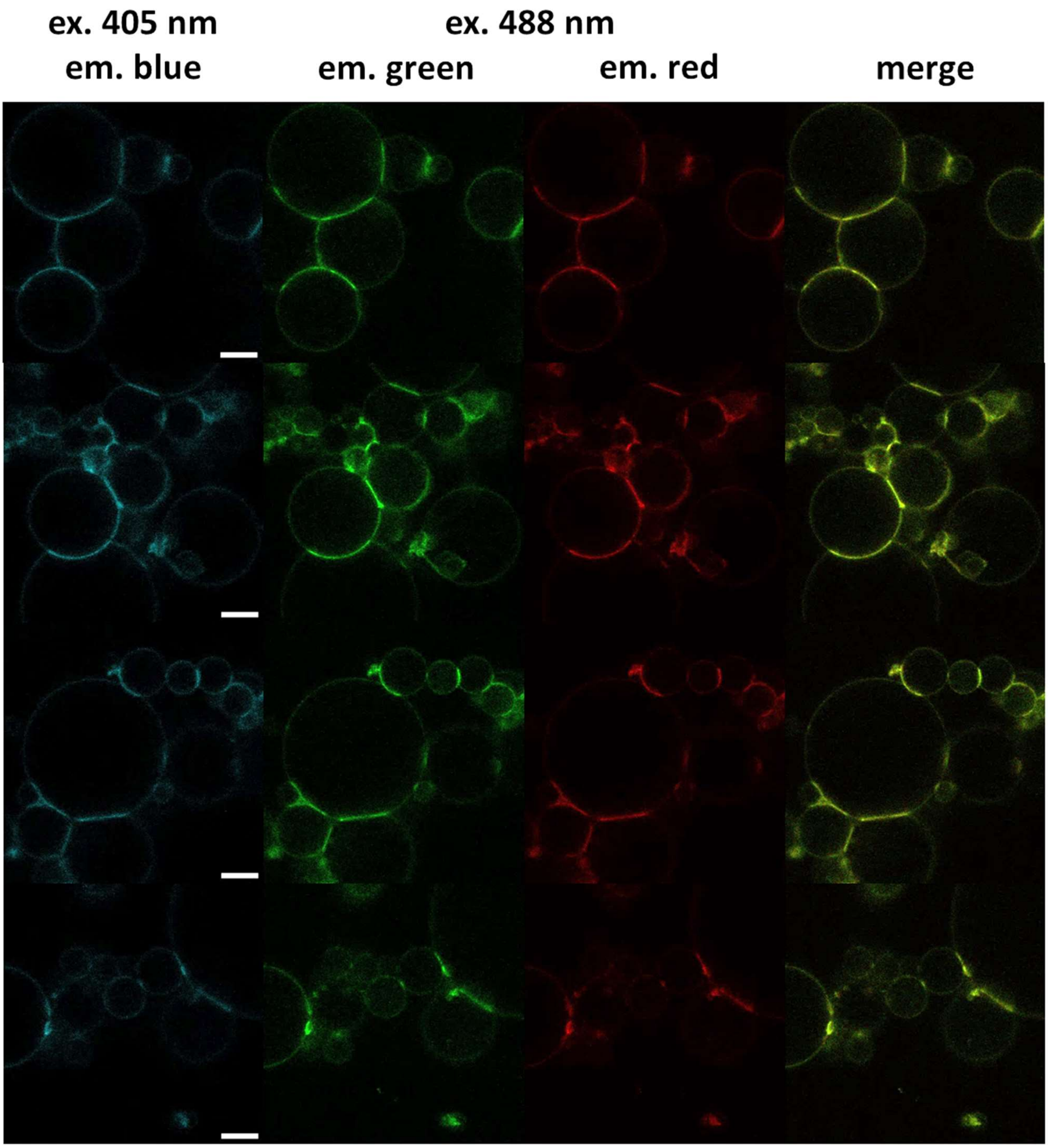


**Figure S33.** Additional fluorescence microscopy images of lectin clustered GUVs presenting $Man_4$DA-Chol after UV irradiation. Scale bars: 10 µm.

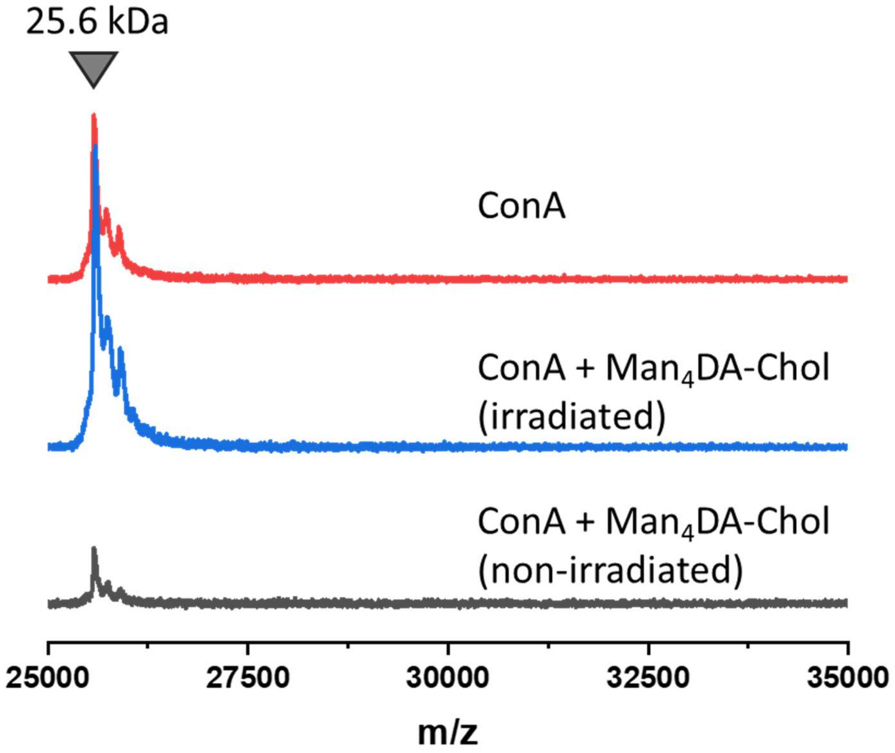


**Figure S34.** MALDI-TOF MS measurements of native ConA (red), ConA + $Man_4DA$-Chol (irradiated)(blue) and ConA + $Man_4DA$-Chol (non-irradiated)(grey).

# References

(1) Ponader, D.; Wojcik, F.; Beceren-Braun, F.; Dernedde, J.; Hartmann, L. Sequence-Defined Glycopolymer Segments Presenting Mannose: Synthesis and Lectin Binding Affinity. *Biomacromolecules* **2012**, *13*, 1845–1852.

(2) Boden, S.; Reise, F.; Kania, J.; Lindhorst, T. K.; Hartmann, L. Sequence-Defined Introduction of Hydrophobic Motifs and Effects in Lectin Binding of Precision Glycomacromolecules. *Macromolecular bioscience* **2019**, *19*, e1800425.

(3) Kim, H. J.; Ishii, T.; Zheng, M.; Watanabe, S.; Toh, K.; Matsumoto, Y.; Nishiyama, N.; Miyata, K.; Kataoka, K. Multifunctional polyion complex micelle featuring enhanced stability, targetability, and endosome escapability for systemic siRNA delivery to subcutaneous model of lung cancer. *Drug delivery and translational research* **2014**, *4*, 50–60.

(4) Song, Z.-J.; Meng, S.-Y.; Wang, Q.-R. Total Synthesis of Marine Alkaloids Motuporamines A and B via Ring Expansion of Cyclic β-Keto Esters. *ACS Omega* **2021**, *6*, 881–888.
